# The influence of different downstream plate length towards the flow-induced vibration on a square cylinder

**DOI:** 10.1038/s41598-023-44388-w

**Published:** 2023-10-17

**Authors:** Nurshafinaz Mohd Maruai, Mohamed Sukri Mat Ali, Sheikh Ahmad Zaki, Jorge Alfredo Ardila-Rey, Izuan Amin Ishak

**Affiliations:** 1 Malaysia-Japan International Institute of Technology, Universiti Teknologi Malaysia, 54100 Kuala Lumpur, Malaysia; 2 Department of Electrical Engineering, Universidad Técnica Federico Santa María, 8940000 Santiago de Chile, Chile; 3 Department of Mechanical Engineering Technology, Faculty of Engineering Technology, University Tun Hussein Onn Malaysia, 84600 Pagoh, Johor Malaysia

**Keywords:** Energy science and technology, Engineering, Mathematics and computing

## Abstract

The investigations of flow-induced vibration have been around for decades to solve many engineering problems related to structural element. In a hindsight of advancing technology of microelectronics devices, the implementation of flow-induced vibration for energy harvesting is intrigued. The influence of downstream flat plate to flow-induced vibration experienced by a square cylinder is discussed in this study to surpass the limitation of wind energy due to geographical constraints and climate change. The mechanism of flow-induced vibration experienced by a square cylinder with downstream flat plate is numerically simulated based on the unsteady Reynolds Navier–Stokes (URANS) flow field. The Reynolds number, *Re* assigned in this study is ranging between $4.2 \times 10^3$–$10.7 \times 10^3$ and the mass damping ratio designated for the square cylinder is $m^*\zeta$ = 2.48. The influence of three different flat plate lengths $w/D = 0.5$, 1 and 3 is examined. Each case of different flat plate is explored for gap separation between the square cylinder and the plate in the range $0.5 \leqslant G/D \leqslant 3$. Based on the numerical findings, the configuration of cylinder-flat plate with length $w/D = 1$ has shown the highest potential to harvest high energy at comparatively low reduced velocity.

## Introduction

The depletion of finite resources of energy is leading to continuous and increasing demand for renewable and sustainable energy. The urge in discovering new advancing technology for practical and cost saving means of renewable energy is crucial at the present. Renewable energy is known for its abundant supply from clean resources with lower environmental impact compared to other conventional energy. Harvesting energy from natural resources contributes to the reduction of carbon dioxide, CO$_2$, therefore reducing the effects of global warming. There are several types of renewable energy resources such as solar, wind, thermal and hydropower^1^. Unlike the non-renewable energy resources, the resources of renewable energy are constantly replenished and dwindled. More importantly, energy harvesting enables exploitation of these resources to empower machinery and electronic devices^2,3^. Apart from the essential natural renewable resources (air,water,solar and biomass), energy harvesting can also be obtained from the aerodynamic instabilities due to fluid loads subjected on structures^4^. This phenomenon is commonly referred as the flow-induced vibration. Due to its sustainability and abundance in environment, this type of vibration has extensive potential to generate an ample electricity for empowering sensor^1^. Unlike the conventional mechanical vibration from machinery and human powered^5^, aerodynamic instability produces vibration from fluid flow energy. Sanchez et al.^6^ found that the micro energy harvesting is practically accessible from the forces generated by the Karman vortex street.

The exploitation of the flow energy by means of wind turbines and watermills to generate electricity have been well developed many years ago^7,8^. Although such technology has been profoundly established and reliable towards the renewable energy sector, there are some concerns particularly regarding the construction cost and design of the generator^8^. The conventional wind turbine is also expected to affect the environment especially towards animals due to the high translational noise from the blade of wind turbine^9^. On another note, the energy harvesting from airflow using wind turbine has been significantly developed and its potential to generate electricity in macro scale is undeniable except for the adverse effect on the environment and climate change. A fair amount of recent studies have work on a small wind turbine as a new paradigm to extract electricity for domestic purpose^10–14^. According to their study, the problematic aeroacoustic noise has not been fully mitigated and this is supported by a survey conducted by Taylor et al.^15^. Based on the previous reports, small scale wind turbine may not be appropriate for micro scale energy harvesting. First, the effect of friction losses in the bearing part may increase due to its reduced size. Consequently, reducing the efficiency to harvest energy. Second, the rotating part of wind turbine, which is expected to experience bearing damage (wear and tear) is not desirable for remote application. Bluff body on the other hand, has more robust features, which could delay the wear and tear issues compared to rotating wind turbine. Energy harvesting using free vibrating bluff body is most likely to be a great alternative for micro scale demand^16^.

Fluid flow energy comes from the interaction of flow by the generated forces with the rigid body when elastically immersed in a flow stream. Rostami and Armandei^8^ classified the vortex-induced motion into the rotation and oscillation categories. Autorotation and second degree of freedom flutter fall onto the rotation type. Whilst, oscillation governs by resonance (VIV, buffet, flutter) and instability motion (flutter and galloping). The approach of harvesting the energy from flow-induced motion in recent years have been associated with vortex-induced vibration, wake galloping, galloping and structural buffeting^17–25^. The flow vibration driven harvester built from a bluff body concept has been well received in the energy community. The idea of harvesting energy was first raised by Duncan^26^ by means of flutter phenomenon. Later, this idea was conceptualized by Mckinney and Delaurier^27^ by adopting the rigid horizontal wing into the windmill. They stated that the wingmill is feasible of producing power efficiently based on series of experiment. The prospect of harvesting wind energy is very well accepted by researchers ever since then. Wings of different shapes, rigid and flapping blades are taken into consideration^28–31^. Flutter, like any other aerodynamic instabilities is considered fatal towards the health of structure due to the cyclic stress and strain^32^. Nonetheless, Dickson^33^ proposed a tree consisting of a collective fluttering piezoelectric leaves as a wind energy harvesting system. Li and lipson^34^ are closely following the proposal of Dickson^33^ by designing a single piezo-leaf. Their piezo stalk-leaf system has evolved by testing different length, orientation and bending stiffness of hinged stalk. Based on the study, the highest peak power is produced by the short stalk of cross-flow configuration^35^. Another plausible work of piezo-element oriented was by Weinstein et al.^18^. Their work exploited the interaction of bluff body wake and a fin to increase and enhance the strain distribution on the piezoelectric bender. The generated power of 100-3000 $\mu$W within velocity of 2-5 ms$^{-1}$ has been successfully produced, which enough to operate a node of sensors^18^.

There are two common transducer mechanisms for kinetic energy based harvester^36^. As reviewed previously, piezoelectric has been widely explored by researchers of flow driven harvester. The relevance of such profound interest is due to the production high power density and high susceptibility to the integrated system^16^. Electromagnetic transducer utilizes the displacement of permanent magnet and coil, which is producing magnetic flux. Magnetic flux is responsible generating the electricity in electromagnetic transducer. Bernitsas et al.^37^ have designed and patented a VIVACE (Vortex-Induced Vibration for Aquatic Clean Energy) converter in 2008. The VIVACE model utilizes the vortex-induced vibration to convert ocean current energy into electricity. The findings have been successful with remarkably high power conversion from fluid flow. For the case of airflow, Jung et al.^38^ have introduced a state of art wind energy harvester from wake galloping phenomenon, which is capable of generating enough electricity for commercial wireless sensor nodes (WSN) platform, iMote2. Based on the published reports from previous experimental studies, the feasibility of energy harvesting from flow-induced vibration has been well demonstrated and conveyed. For reference, the flow energy harvesting system investigated by previous studies are summarized in Table 1. Due to the increasing demand of automated electronic appliances, as well as the growth of technology evolution, the interest in exploiting the sustainable resource such as the flow-induced vibration for a further development is anticipated. The reinforcement for further investigation in regards of its stability and applicability is necessary. One of many ways to improve the harnessable energy from the flow-induced vibration is by employing the flow control strategy towards the bluff body of concern^39^.

**Table 1:** Energy harvesting system associated with flow-induced vibration.

Authors	Medium	Transducer	Output power	Application
	Flow speeds		(mW)	
Jung and Lee^38^	Air	Electromagnetic	370	Bridge monitoring system
	0–22.5 ms$^{-1}$			
Weinstein et al.^18^	Air	Piezoelectric	3	Piezo-tree generator
	2-5 ms$^{-1}$			
Li et al.^35^	Air	Piezoelectric	0.6	Heating, Ventilation and Air Conditioning (HVAC) system
	0.3-8 ms$^{-1}$			In smart building
Liu et al.^40^	Air	Piezoelectric	1.06	Wireless sensor nodes (WSN)
	0-10 ms$^{-1}$			In remote area
Koide et al.^20^	Water	Electromganetic	10	River monitoring system
	2-25 ms$^{-1}$			
Bernitsas et al.^37^	Water	Electromagnetic	10$^{6}$	VIV aquatic clean
	1.5 ms$^{-1}$			Energy converter

### Flow control method as an enhancer of flow energy

In engineering application, the common approach to overcome uncertainties regarding bluff body in fluid flow is by using control method. In general, the control of flow over a bluff body can be classified into active and passive control^39^. The active control requires external mechanisms like powered actuator to change the flow structures, while passive vibration control is an external geometry modification. The passive control modifies the flow structure over a bluff body by means of controlling the boundary layer separation or wake of the bluff body^41^. The application of the control method is highly dependent on the geometry of a bluff body. For a circular cylinder or sphere, the boundary separation point is different and changing based on Reynolds number. Distinctively, the blunt bluff body usually has a fixed separation point, which exhibits sudden change of boundary layer into the near wake flow. Choi et al.^41^ classified the control strategies into boundary layer control and direct wake control. The first method (boundary layer control) curated for a bluff body with a curvature and no fixed separation point (i.e. circular cylinder) and conversely, the latter method suitable for a bluff body with or without fixed separation. The surface roughness or dimple on bluff body was initially applied to delay the separation of boundary layer and consequently reduces the drag force and wake formation.

In the light of harvesting the energy from flow-induced vibration (FIV) phenomena, the boundary layer control method has been investigated by an established team of researchers in Michigan University^37^. Contrarily to the theory of control, the surface roughness on a circular cylinder expedited the vortex generation and maintains the increasing amplitude of vibration. Chang et al.^21,42^ found that the location of roughness strip profoundly affects the cylinder vibratory response. The roughness strips expedites the vortex generation and maintains the increasing amplitude of vibration at its effective location. Experimental investigations by Chang et al. have reported that the effective location of surface roughness for energy harvesting is ranging between 20$^o \leqslant \alpha _{PTC} \leqslant$ 64$^o$. Nevertheless, this control method is only accessible for a bluff body with curvature and no fixed separation point.

The other prominent passive control method is direct wake control method. Splitter plate is commonly assigned downstream to a bluff body to alter the interaction of alternating vortices in the wake of a bluff body. Unlike the boundary layer control strategy, this method compromises the flow control of a bluff body with sharp edged and fixed separation points. The investigations of wake control utilizing splitter plate have been done by aerodynamists^43–47^. According to Chan^47^, the efficiency of this wake control method is highly influenced by the length of splitter plate and the gap distance between that splitter plate and a bluff body. Two flow regimes were found by Ali et al.^46^ with critical gap separation of $G_{c}/D$ = 2.3 for a rigid body at low reynolds number. Samion et al.^48^ also identified the same regimes for a wedge as the wake passive control with critical gap of $G_{c}/D$ = 2.76 for $Re = 22 x 10^3$.

The first regime is characterized by the reattachment of separated shear layer from the cylinder in the wake of plate or wedge. The second regime is observed when the recirculation of shear layers is within the gap separation between cylinder and plate or wedge. The vortex formation is accomplished in this regime and thus, results in the return of the aerodynamic forces^46^ and sound^48^ towards the vicinity of a single square cylinder. For an oscillating body, these regimes were introduced by Unal and Rockwell^49^ as pre-formation and post-formation regimes. Numerical experiment by Chan et al.^47^ discovered the two flow regimes with critical gap separation of $G_{c}/D$ = 2.5. In correspondence to the terminology introduced by previous studies, the pre-formation vortex regime of which the interference of splitter plate is effective is found when the gap separation is ranging 0 $\leqslant G/D \leqslant$ 2.5 (square cylinder) and 0 $\leqslant G/D \leqslant$ 2 (circular cylinder). Based on previous reports of direct wake control, the first flow regime is anticipated to be favourable for energy harvesting purpose.

Direct wake control method has also been implemented by researchers due to energy harvesting application as a mean to increase the harvested energy and expand the working velocity of a harvester. By exploiting the wake galloping principle, Jung and Lee^38^ have successfully generated 370mW under wind speed condition 4.5 ms$^{-1}$. The configuration of two cylinders in tandem arrangement at gap distance 5D enables to power an Imote2 sensor for sensing process in bridge monitoring system. Another similar example was investigated by Koide et al.^20^ for river monitoring system. The cylinder-plate in cruciform arrangement was utilized and resulted in expansion of working velocity for the harvester in changing river flow.

### The selection of flat plate length

Due to the rising demand of harnessable energy from the environment, this study attempts to investigate the potential of a passive flow and vibration control to enhance the flow-induced vibration particularly from airflow and simultaneously increase the amount of generated power. Moreover, the limitation of low wind speed in tropical climate countries such as Malaysia also motivates the exploitation of the passive vibration control. In pursuance of surpassing the disadvantages of low wind speed in Malaysia and ultimately harnessing the wind energy into usable electricity, a downstream flat plate is introduced in this study. According to Ali et al.^46^ the presence of a splitter plate located downstream to the bluff body generates remarkable change in the flow characteristic and wake structure of the upstream bluff body. Therefore, a downstream flat plate is introduced in this work to evaluate the possibility of enhancing the vibration amplitude for energy harvesting purpose.

**Figure 1:**
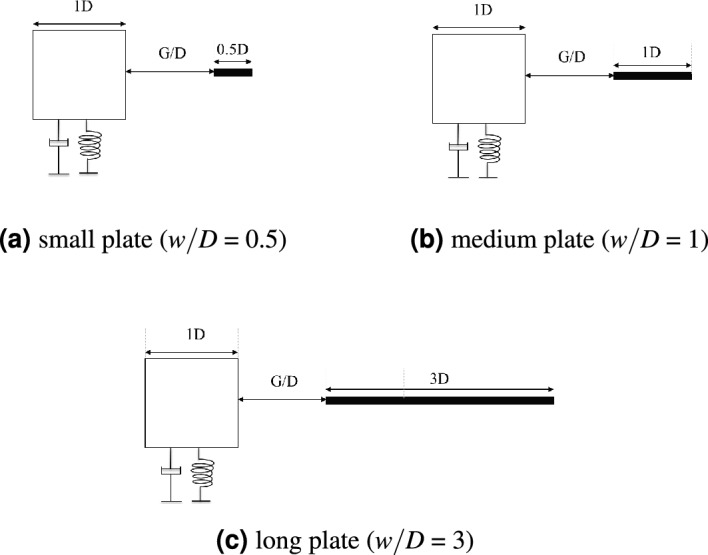
Configurations of plate length variables.

Changes in flow characteristic of a bluff body can be influenced by the variation of a downstream splitter plate length L/D. The effect of downstream splitter plate has been studied comprehensively by Apelt et al.^50^ regarding the splitter plater of 0.5 $\leqslant L/D \leqslant$ 2 and followed by the second part of the study associated with long splitter plate^51^. Based on their findings, the short splitter plate remarkably altered the properties of flow over the bluff body, where the alternate fluctuating vortices were apparent until L/D = 2. While, no changes were observed for splitter plate L/D $\geqslant$ 5 (circular cylinder) and splitter plate L/D $\geqslant$ 3 (normal flat plate)^51^. Furthermore, different types of bluff body geometries have been investigated and unanimously acknowledged the dependency of wake interference to the selection of plate length^44,52,53^. In light of the findings from previous studies, three flat plate lengths with thickness $h_{p}/D$ = 0.1 as illustrated in Fig. 1 were investigated. Flat plates with length *w*/*D* = 0.5, 1 and 3 were considered and addressed as small plate, medium plate and long plate respectively.

According to previous investigation by Apelt et al.^50,51^, the drag and vortex shedding in the wake of a normal flat plate with fixed separation points is significantly modified with the presence of a splitter plate of length 0 $\leqslant w/D \leqslant$ 3. However, it is noteworthy that in these investigations, the gap separation between the upstream body and downstream body is absence (*G*/*D* = 0). Henceforth, the selection of flat plate length variable is adapted with the minimum predetermined gap separation of G/D = 0.5 between a square cylinder and flat plate. In this study, L/D is redefined as the gap distance between the downstream of cylinder towards the rear end of flat plate. Wake alteration caused by the reduction of drag coefficient and the increased of base pressure is remarkable when a splitter plate of length $L/D \leqslant$ 2^50^. Therefore, in this study, flat plate of length *w*/*D* = 0.5 (*L*/*D* = 1) and *w*/*D* = 1 (*L*/*D* = 1.5) were selected to assess the trend of change that occurs in the wake of square cylinder when the gap separation between the cylinder and plate is varied. Whilst, flat plate of length *w*/*D* = 3 (*L*/*D* = 3.5) is considered in this study to verify its ineffectiveness towards the wake of a square cylinder as suggested in previous study^50^.

## Methodology

### Model and computational domain description

The main interest of geometry in the current study is a square cylinder with side length *D*. Square cylinder is accounted as the principal body for the wind harvester. This generic and simple geometry is opted as the baseline case due to its wide application in engineering structures^54^. However, the properties flow field around the square cylinder is rather complicated as compared to a circular cylinder. The challenge is always associated with the flow phenomena such as flow impinging, separation and also the response of vortex shedding in the wake [130]. This conflict usually leads and resulted in small discrepancies of flow analysis. Therefore, many efforts have been made to analyse the flow structure and its properties by conducting numerical simulation^55–60^.

In present study, for an isolated single square cylinder case, the dimension of computational domain is 31D x 21D (width x height)^61^. The outlet was determined relatively far (20D) from the body to eliminate the effect of end wall and to opt for real case scenario^58^. Similar computational domain size was also opted by simulation works of rigid body by Doolan^57^, Ali et al.^54^, and Samion et al.^48^. In like manner, the simulation work of vibrating body by Pan et al.^62^ also employed outlet boundary 20D from the circular cylinder. These work have demonstrated good and reliable results and proved unnecessary further extension of the size of computational domain.

The computational domain is constructed based on the near body gradient mesh similar to previous study by Ali et. al^54^. All grid parameters associated with this current study are summarized in Table 2. The $y^+$ values of the three mesh grids are compared with the value of $y^+$ from previous studies. The study by Murakami et al.^63^ investigated the occurrence of flow-induced vibration of a square cylinder while Samion et al.^48^ adapted a square cylinder geometry with similar mesh distribution as in this study. The $y^+$ value of fine mesh is in a very good agreement with the findings from both studies.

**Table 2:** Grid parameters for grid refinement study.

Case	A (Fine)	B (Medium)	C (Coarse)	Samion et al.^48^	Murakami et al.^63^
Total No. of cells, *N*	101,662	56,316	31,036	108,018	7,176
No. of cells near cylinder, $N_\text {C}$	144	84	64	–	–
Average cell size, *h*/*D*	0.1857	0.2261	0.2758	–	–
Average $y^+$	11.17	18.56	29.74	11.26	11.81

A grid convergence study was also performed to confirm the credibility of the numerical simulation around the square cylinder is validated. Based on previous studies, the accuracy of numerical simulation can be retrieved when the error of grid dependence is minimized^54,64^. Table 3 shows the results of order accuracy and the GCI value of three resolution grids. An evident reduction of GCI value was observed for finer grid resolution when compared with coarser order grid resolution (GCI$_{21}$ < GCI$_{32}$). The GCI value for finer grid resolution had improved from the initial GCI value of coarser grid resolution, hence reduced the influence of cell size to the numerical simulation. As the GCI was decreased significantly for finer grid resolution compared to coarser grid, the grid independent solution was assumed to be practically accomplished. Thus, any further refinement of grid resolution has very little or no effect on the simulation^54^.

**Table 3:** Order of accuracy and the grid convergence index (GCI) for various parameters of different reduced velocity.

Reduced velocity, $U_{R}$	Parameters	$\mid \varepsilon _{21}\mid$	$\mid \varepsilon _{32}\mid$	*R*	GCI$_{21}$ (%)	GCI$_{32}$ (%)
6	$y_t{rms}$/D	0.0001	0.0002	0.5000	0.6843	5.1730
	$f/f_{n}$	0.0084	0.0127	0.6614	0.3709	1.7790
	$C_{Lrms}$	0.0063	0.0685	0.0920	0.0038	0.5045
8.5	$y_{rms}$/D	0.0015	0.0041	0.3659	0.8424	10.9367
	$f/f_{n}$	0.0094	0.0172	0.5465	0.2005	1.2847
	$C_{Lrms}$	0.0373	0.2533	0.1473	0.0543	3.1595
18	$y_t{rms}$/D	0.0023	0.0065	0.3538	0.5456	9.8304
	$f/f_{n}$	0.0013	0.0063	0.2063	0.0053	0.1681
	$C_{Lrms}$	0.0270	0.0641	0.4212	0.2931	2.8639

**Figure 2:**
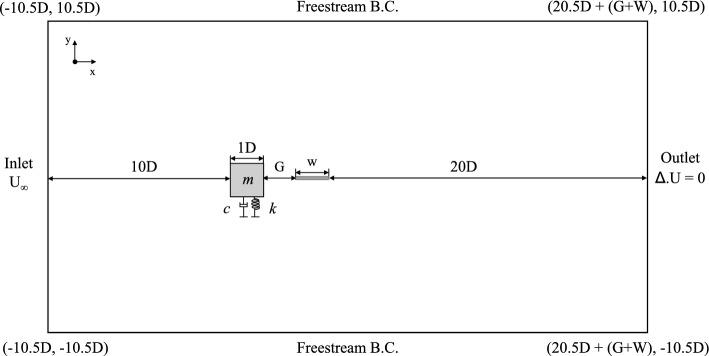
Schematic diagram of the computational domain and boundary conditions.

In this study, the cylinder-plate configuration is incorporated within the computational domain as depicted in Fig. 2. The gap separation between the square cylinder and the flat plate was changed from 0.5 $\leqslant G/D \leqslant$ 3. The number of cells between square cylinder and downstream plate varies according to the changes of gap separation to secure the cell size around the cylinder whereas the cells behind the plate are organized by gradient method^54^. In general, the size of computational domain in this study is changing due to the variation of plate length and its corresponding gap separation between the square cylinder and downstream plate.

The inlet was designated with fixed freestream reduced velocity and the outlet was set with zero gradient. The upper and lower walls were set as free stream boundary with no slip boundary condition of distance 10D from the edge of the square cylinder. According to the computational domain illustrated in Fig. 2, the boundary condition set up is summarized in Table 4.

**Table 4:** Boundary condition set up.

Patch/Boundary	Inlet	Outlet	Square	Plate
*U* (m/s)	fixedValue	zeroGradient	movingWallVelocity	fixedValue
*p* (Pa)	zeroGradient	fixedValue	zeroGradient	zeroGradient
*k* (m$^2$/s$^2$)	fixedValue	zeroGradient	kqRWallFunction	kqRWallFunction
$\omega$ (s$^{-1}$)	fixedValue	zeroGradient	omegaWallFunction	omegaWallFunction
*nut* (m$^2$/s)	fixedValue	zeroGradient	nutkWallFunction	nutkWallFunction

### Numerical approach

#### Flow solver

The flow is modelled according to the conversion law of mass and momentum in predefined volume. Flow simulations are conducted utilizing an open source of CFD solver, OpenFOAM that is oriented based on C++ libraries. The flow solutions are obtained by solving the continuity and incompressible Reynolds Averaged Navier–Stokes (RANS) equations for an incompressible flow are given as follows;

1 $$\frac{{\partial u_{i} }}{{\partial x_{i} }} = 0$$

2 $$\begin{aligned}{} & {} \quad \rho \frac{\partial \overline{u_i}}{\partial t}+\rho \overline{u_j}\frac{\partial \overline{u_i}}{\partial x_j} = \frac{\partial }{\partial x_j}\left[ -\overline{p}\delta _{ij}+\mu \left( \frac{\partial \overline{u_i}}{\partial x_j}+\frac{\partial \overline{u_j}}{\partial x_j}\right) -\rho \overline{u'_i u'_j}\right] \end{aligned}$$

Here $\overline{u}$ is the time averaged velocity. In addition to the discretized flow solution, the turbulence model employed for this work is SST ($k-\omega$) in order to determine the Reynolds stress tensor and simultaneously solve the governing equations.This solver has been reported to be computational time efficient and reliable for the study of flow-induced vibration on a bluff body^65, 66^. Reynolds stress tensor, $\tau _j = \rho \overline{u'_i u'_j}$ is a turbulence transport term, which address the fluctuating velocities to the total change in conservation of momentum. Subscripts *i* and *j* represent the tensor index notation of two dimensional Cartesian coordinate. The Reynolds stress tensor is solved using the Boussinesq assumption.

3 $$\begin{aligned} -\rho \overline{u'_i u'_j} = \mu \left( \frac{\partial \overline{U_i}}{\partial x_j}+\frac{\partial \overline{U_j}}{\partial x_i}\right) \end{aligned}$$

The turbulence properties of the flow are decomposed on turbulent kinetic energy *k* and turbulent dissipation rate $\omega$. The equations are given by;

Turbulent kinetic energy *k*

4 $$\begin{aligned} \frac{D\rho k}{Dt} = \tau _{ij}\frac{\partial u_i}{\partial x_j} - \beta ^*\rho \omega k + \frac{\partial }{\partial x_j} \left[ \left( \mu + \sigma _k \mu _t\right) \frac{\partial k}{\partial x_j}\right] \end{aligned}$$

Turbulent dissipation rate $\omega$

5 $$\begin{aligned} \frac{D\rho \omega }{Dt} =\tau _{ij} \frac{\gamma }{\nu _t} - \beta \rho \omega ^2 k + \frac{\partial }{\partial x_j} \left[ \left( \mu + \sigma _\omega \mu _t\right) \frac{\partial \omega }{\partial x_j}\right] +2\rho (1-F_1)\rho \sigma _{\omega 2} \frac{1}{\omega }\frac{\partial k}{\partial x_i} \frac{\partial \omega }{\partial x_j} \end{aligned}$$

Here $\nu _T$ is kinematic viscosity and given as

6 $$\begin{aligned} \nu _T =\frac{a_1 k}{\max (a_1 \omega ,\Omega F_2)} \end{aligned}$$

$\Omega$ represents the absolute value of vorticity while *F*$_2$ is the closure coefficient indicating the boundary flows and described as:

7 $$\begin{aligned} F_2 = \tanh \left[ \left[ \max \left( 2\frac{\sqrt{2}}{0.09\omega y},\frac{500 \nu }{y^2 \omega }\right) \right] ^2 \right] \end{aligned}$$

Another closure coefficient *F*$_1$ is formulated as follows:

8 $$\begin{aligned} F_1= \tanh \left[ \min \left[ \max \left( 2\frac{\sqrt{k}}{0.09\omega y},\frac{500 \nu }{y^2 \omega }\right) ,\frac{4 \rho \sigma _{\omega 2}k}{CD_{k\omega }y^2}\right] ^4 \right] \end{aligned}$$

Here *y* is the distance to the next surface and *CD*$_\omega$ attends to the positive part in diffusion term of Eq. (5);

9 $$\begin{aligned} CD_{k\omega } = \max \left( 2\rho \sigma _{\omega 2} \frac{1}{\omega }\frac{\partial k}{\partial x_j}\frac{\partial \omega }{\partial x_j},10^{-20}\right) \end{aligned}$$

The constants of $\phi$ for SST ($k-\omega$) is derived as given in Eq. (10) and using following parameters;

10 $$\begin{aligned} \phi= & {} \phi _1 F_1 + \phi _2 (1-F_1) \end{aligned}$$

11 $$\begin{aligned} \beta _1= & {} \frac{3}{40}, \quad \quad \quad \quad \beta _2 = {0.0828}, \quad \quad \quad \quad \beta ^* =\frac{9}{100} \end{aligned}$$

12 $$\begin{aligned} \sigma _{k1}= & {} 0.85, \quad \quad \quad \quad \sigma _{k2} =1, \quad \quad \quad \quad \sigma _{\omega 2}=0.856 \end{aligned}$$

The governing equations are solved by utilizing the OpenFOAM CFD software. The merged PISO (Pressure Implicit Split Operator)- SIMPLE (Semi-Implicit Method for Pressure-Linked Equations) algorithm, PIMPLE is used as the pressure–momentum coupling in one time step twice to resolve the problem in this study. In general, the Courant-Fredichs-Lewy (CFL) number is required to be less than 1 to provide a stable simulation and accurate solution. According to previous study on flow-induced vibration on bluff body, the acceptable range for CFL number is between 0.7 to 1^65,66^. In the current study, for a single isolated cylinder case, the non-dimensional time step is set to 0.001. For each succeeding case, the non-dimensional time steps is set sufficiently to obtain stable solutions by the CFL number is being kept at 0.8.

The fluid-structure kinematic problems are solved according to the finite-volume discretization method (FVM). Based on Taylor expansion formulation, the temporal term is discretized by second order backward differencing scheme while the convection term of transport equations is discretized using the third order of backward differencing QUICK scheme of non-uniform grid. The diffusivity term is discretized into second order unbounded Gauss linear differencing scheme. In order to retrieve second order of accuracy, the source term is discretized by integration of control volume based on the Gauss’ theorem. The non-linearity of momentum equations is addressed in pressure-velocity coupling algorithm and in this particular work the PIMPLE algorithm is utilized. The summary of the discretization procedure of the transport equation and numerical scheme is presented in Table 5.

**Table 5:** Summary of numerical and discretization method.

Discretization	Scheme
Temporal discretization	2nd order backward scheme
Convection term	3rd order QUICK scheme
Viscous term	2nd order unbounded Gauss linear differencing scheme
Pressure-velocity coupling	PIMPLE
Turbulence model	SST ($k-\omega$)

#### Dynamic mesh motion

As the main purpose of this work is to assess the characteristic of flow-induced vibration, it is essential to operate with a solver that allows dynamic mesh formation. OpenFOAM package offers the PIMPLE solver, which complement the needs of transient flow with moving mesh. Jasak and Tukovic^67^ developed an automatic mesh motion algorithm to adequately determine the motion of moving mesh. The mesh deformation is resolved by point position update and the mesh is allowed to deform automatically based on the Laplacian smoothing equation given by;

13 $$\begin{aligned} \ \nabla .(\gamma \nabla u)=0 \end{aligned}$$

where *u* is the mesh deformation velocity of the nodes in the mesh and $\gamma$ is displacement diffusion,where a square of the inverse of the cell volume;

14 $$\begin{aligned} \gamma = \frac{1}{l^2} \end{aligned}$$

where *l* is the cell centre distance to the nearest selected boundary, i.e., square cylinder edges) has been chosen for this study. Therefore,the cell distortion is higher only when the cell distance from the cylinder surfaces is far and the cell quality near the square cylinders is preserved. Generalised Geometric-Algebraic Multi-Grid (GAMG) linear solver with Gauss Seidel smoother is used to solved iteratively Eq. (13). The tolerance for the iteration to complete is $1\times 10^{-9}$. The mesh deformation velocity is used to update the new point positions of the mesh distributions:

15 $$\begin{aligned} x_{new} = x_{old} + u\Delta t \end{aligned}$$

The transverse motion experienced by the square cylinder is simulated according to the automatic mesh motion algorithm following Jasak and Tukovic^67^. From the numerical perspective, the motion of the square cylinder is captured from the deformation of grid mesh based on the Laplacian smoothing equation. All parameters associated with the motion of square cylinder in this numerical study correspond to the previous wind tunnel testing by Maruai et al.^68^.

In order to generalize the problem, non-dimensonalized parameters were introduced and summarized in Table 6. These parameters were derived from a single degree of freedom (SDOF) elastic system. The structural parameters were curated into dimensionless, by governing the natural frequency $f_\text {n}$ and side length of cylinder *D* into unity. The Reynolds numbers, *Re* assigned in this study were ranging between 4.2 x 10${^3}$ to 10.7 x $10{^3}$. This subcritical range implies to the fluid flow condition adopted for energy harvester used in the remote area. These harvesters are widely applied in structural health monitoring system^22,38^ and unmanned vehicle^69^. Findings from previous studies have shown that a significant amount of power can be harvested for wind velocity ranging between 3 ms$^{-1}$–7 ms$^{-1}$^22–25,70^. Taking that range into consideration, the present numerical studies were conducted in similar range of Reynolds number derived from reduced velocity, $U_R$, side length of cylinder, *D* and kinematic viscosity, $\nu$.

**Table 6:** Non-dimensional parameters for mesh moving algorithm.

Parameters	Symbol	Definition	Magnitude
Mass ratio	$m^*$	$\displaystyle \frac{m}{\rho D^2L}$	566.74
Damping factor	$\zeta$	$\displaystyle \frac{\delta }{2\pi }$	0.0043767
Reduced velocity	$U_{R}$	$\displaystyle \frac{U}{f_n D}$	5–20
Reynolds number	Re	$\displaystyle \frac{\rho UD}{\nu }$	(4.2–10.7) $\times$ $10^{3}$

### Validation study of vibrating model

**Figure 3:**
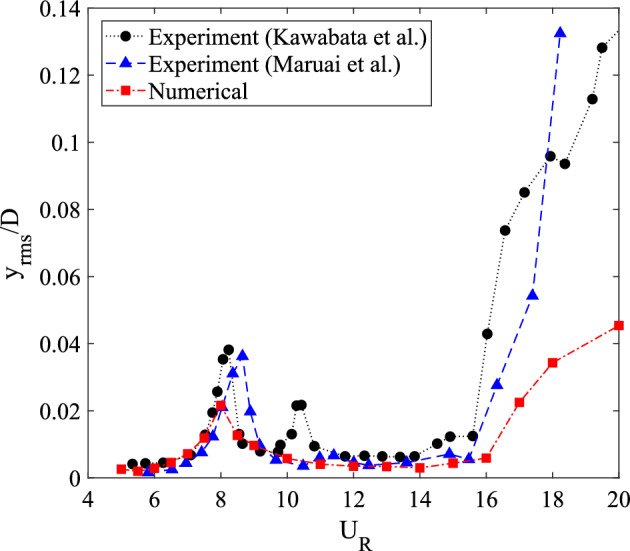
Comparison of amplitude response between numerical and previous studies by Kawabata et al.^71^ and Maruai et al.^68^.

The fine grid resolution was made into a benchmark of the current numerical simulation. Series of numerical simulations were conducted for reduced velocities 5 $\leqslant U_R \leqslant$ 20. This range of reduced velocity is corresponding to wind velocity 2 ms$^{-1} \leqslant U \leqslant$ 7.5 ms$^{-1}$ with the natural frequency of cylinder is $f_n$ = 14.3 Hz and side length of *D* = 0.026 m. The vibration amplitude response of this current numerical simulation is plotted in Fig. 3 along with the previous experimental measurements^68,71^. The trend of vibration amplitude obtained from the numerical simulation is comparable to the vibration amplitude trend reported by the previous studies. The credibility and reliability of two-dimensional numerical simulation to predict the flow-induced vibration for intermediate Reynolds number is convinced by the coinciding trend of vibration amplitude from the current numerical simulation and the existing experimental results.

As shown in Fig. 3, the occurrence of VIV was well observed within the low reduced velocity region, while high amplitude galloping was discovered at the high reduced velocity region. There were two distinct branches of VIV found through the amplitude curve from $U_R$ = 5 to $U_R$ = 14. Based on the terminology by Khalak and Williamson^72^, the initial branch of VIV began at $U_R$ = 5 and followed by the lower branch of VIV between 9 $\leqslant U_R\leqslant$ 14. The lock-in of synchronization character was found intervening between the initial and lower branch of VIV. The increasing trend of vibration amplitude for $U_R\geqslant$ 15 indicating the galloping phenomenon as suggested by Kawabata et al.^71^ was also revealed in Fig. 3.

**Figure 4:**
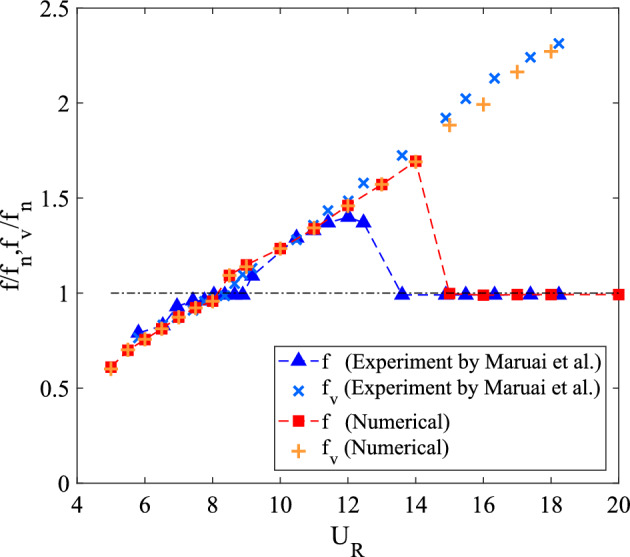
Comparison of frequency response between numerical and experimental measurement^68^.

The dominant frequency of vibration and vortex shedding was determined by the FFT equation and normalized by the natural frequency of cylinder, $f_n$. Figure 4 shows the response of normalized frequency of vibration and vortex shedding over a variation of reduced velocities. The frequency of vibration and frequency of vortex shedding were steadily approaching to unity during initial branch of VIV, of which indicating the lock-in synchronization of VIV. As the velocity further increased, the frequency ratio curve ascending up until $U_R$ = 14 and followed by the sudden drop of frequency of vibration. The plunge of frequency of vibration at $U_R$ = 15 conveyed the dissociation of the vibration character from the influence of vortex shedding. The self-induced mechanism of galloping was evident when $f = f_n \ne f_v$. This confirmed the starting point of galloping at $U_R$ = 15 with an increasing rms vibration amplitude as depicted in Fig. 3. Through this comparison and validation study, it is proven with confidence that a 2D simulation is reliable to simulate the fluid structure interaction and consequently enabling the exploration of flow-induced vibration mechanism of a square cylinder. This is consistent with findings from previous studies^55,56,73^, which investigating the flow-induced vibration at subcritical Reynolds numbers region.

**Figure 5:**
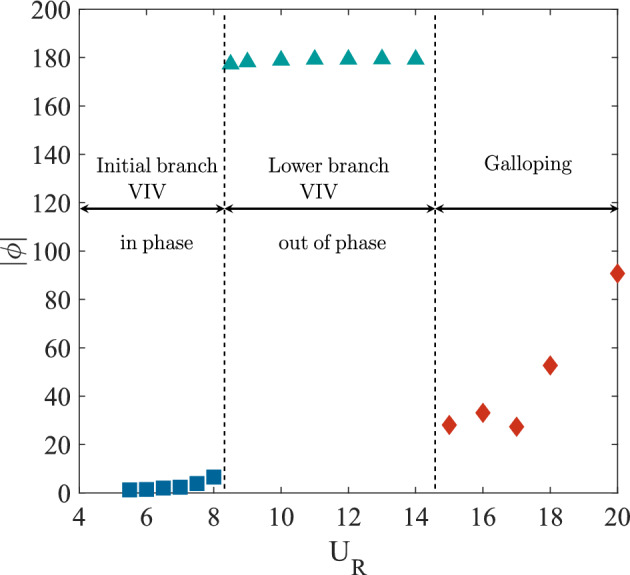
Phase lag and the regimes associated with the FIV of an isolated square cylinder.

Further observation of FIV of square cylinder was made through a phase lag analysis for reduced velocities in the range 5 $\leqslant U_R \leqslant$ 20. Phase lag is the time lag between fluctuating lift force and vibration amplitude retrieved by interpolating both signals in the same time domain. The time lag between the peaks of each signal is measured and by means of FFT the phase lag is determined. Three regimes of FIV behaviour were identified based on the phase lag response in Fig. 5. Adapting the terminology from previous study, the first regime can be classified as the initial branch of VIV^74^. In this regime, the phase lag between vibration amplitude and the lift force coefficient was increased with velocity but still remain close to 0$^{\circ }$. This regime was also corresponding to the increased of amplitude and frequency response when the reduced velocity ranging between 5.5 $\leqslant U_R \leqslant$ 8.5. Following that, an intermittent switch of phase lag from $\phi$ $\approx$ 0$^{\circ }$ to $\phi$ $\approx$ 100$^{\circ }$ occured between the initial and lower branch of VIV at reduced velocity $U_R$ = 8.5. The same switching mode was also observed in Khalak and Williamson^72^.

The second regime reveals the suppression of vibration amplitude and is identified as the lower branch of VIV. The lower branch of VIV were discovered at reduced velocity ranging between 8.7 $\leqslant U_R \leqslant$ 14. During the lower branch of VIV, the lag between the motion of cylinder and the fluctuating lift force was out of phase and persistent near to 180$^{\circ }$. According to Guilmineau and Queutey^75^ the sudden jump of phase lag is associated with the suppression of vibration amplitude as presented in Fig. 3. An increasing pattern of phase lag was observed between 30$^{\circ }$ to 100$^{\circ }$ for reduced velocity $U_R \geqslant$ 15 and it is therefore, confirms the mechanism of galloping in the third regime. According to Nikitas and Macdonald^76^, for dry galloping occurrence, the phase lag between the displacement of cylinder and the lift force coefficient is depicted in the proximity of $\phi$ $\approx$ 60$^{\circ }$.

**Figure 6:**
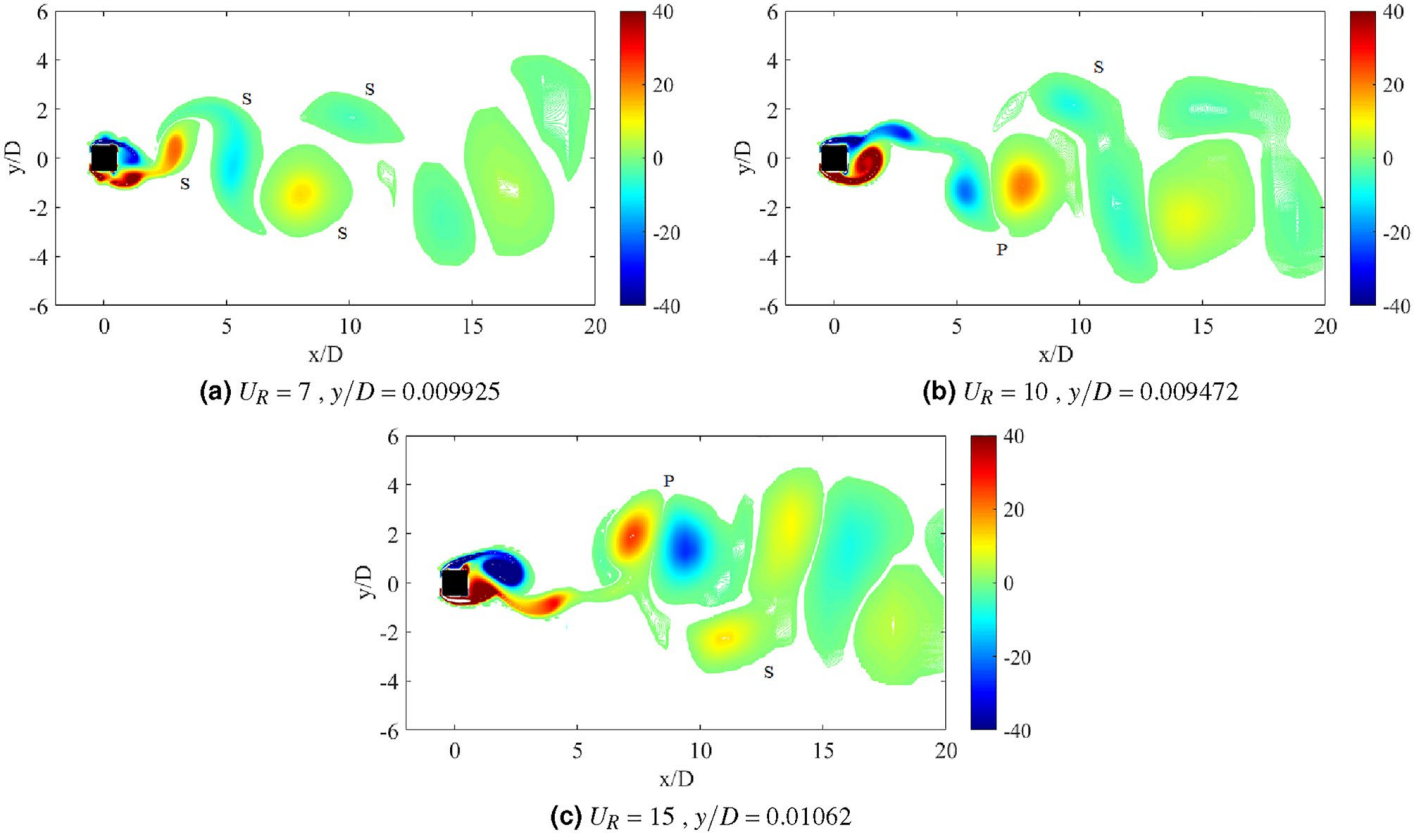
Vortex pattern at different reduced velocities for isolated square cylinder. The symbol $\Omega$ represents the spanwise vorticity and $U_\infty$ = $U_R f_v D$ is the streamwise velocity.

Another important property of flow-induced vibration is the local vorticity distribution. According to Ding et al.^73^, the vibration amplitude and frequency characters are highly influenced by the vorticity distribution. Vorticity distribution produces the vortex patterns in the near wake of cylinder. Williamson and Roshko^77^ revealed a set of vortex pattens in the synchronization regions. Each vortex pattern demonstrates the character of flow near the wake of cylinder. A better understanding on the interaction of wake structure and the motion of square cylinder can be substantiated through the flow visualization of vortex pattern in the wake of cylinder. The vortex patterns established at the maximum amplitude of vibration are illustrated in Fig. 6.

Figure 6a illustrates the alternating single vortices 2S discovered downstream to the cylinder at reduced velocity $U_R$ = 7, of which belongs to the initial branch of VIV. The transition from 2S pattern to P+S pattern was observed due to the further increased in velocity^77^. A well developed P+S vortex pattern was established in the proximity of lower branch VIV at $U_R$ = 10 as shown in Fig. 6b. Besides that, P+S vortex mode was also observed at $U_R$ = 15, which is enclosed in the galloping region. The vortex shedding pattern presented in Fig. 6c and its corresponding frequency are contradicted from the frequency of vibration ($f/f_n$ = 1) found in Fig. 4. Henceforth, the vortex shedding has no significant towards the mechanism of motion experienced by the cylinder^73^. The occurrence of self-induced galloping phenomenon is attested starting from $U_R$ = 15 for a square cylinder.

### Estimation of power from flow-induced vibration

A mathematical model of vibrating system is used to estimate the harvested power in this study. This approach was first suggested by Bernitsas et al.^37^ for the novel hydropower generator or well-known as Vortex Induced Vibration Aquatic Clean Energy (VIVACE). Given that the physical parameters of oscillating system were determined based on the experiment, the same method was applied by Ding et al.^73^ to calculate the harnessable power from flow simulation. The power obtained by calculation, which was plunged with the vibration amplitude and the corresponding frequency of vibration, has shown a good agreement to the power measured from the experiment.

**Figure 7:**
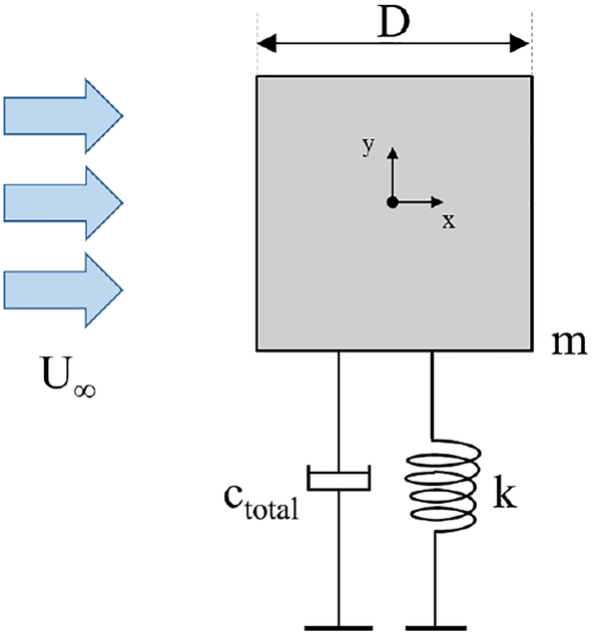
Elastic system of a square cylinder.

Flow-induced vibration is practically modelled as a single degree of freedom (SDOF) elastic system as shown in Fig. 7. The dynamic response of this elastic system due to the fluid force is described by the equation of motion and presented by the second order of linear equation in Eq. (16).

16 $$\begin{aligned} F_y = m{\ddot{y}}+c{\dot{y}}+ky \end{aligned}$$

where y is the direction of cylinder motion transverse to the incoming flow and $F_y$ is the total force exerted on the cylinder from airflow in y-direction, m is the corresponding mass, c is the damping coefficient, and k is the spring stiffness.

The exhibited motion due to the excitation of the fluid force when frequency of vortex shedding $f_v$ is approaching near to the natural frequency of cylinder $f_n$ can be expressed by;

17 $$\begin{aligned} y(t) = y_0 \sin (\omega t) \end{aligned}$$

Here $y_0$ is the maximum cylinder displacement and $\omega$ = 2$\pi$f is the angular frequency for oscillation, whilst f represents the frequency of oscillation. Hence the energy induced from the fluid flow into the elastically mounted cylinder can be described as the work performed to the cylinder by fluid force $F_y$. Such behaviour can be described by the following equation;

18 $$\begin{aligned} W_y = \int _{0}^{T} F_y \, \dot{y} \;\text {dt} \end{aligned}$$

The power produced by the the work done during a cycle of period *T* vibration^20^ can also be defined as the mechanical power extracted from the flow by vibrating cylinder $P_y$^73^.

19 $$\begin{aligned} P_y = \frac{1}{T}\int _{0}^{T}F_y \,\dot{y} \; \text {dt} \end{aligned}$$

Under resonant condition, Eq. (17) is plunging into the power equation in Eq. (19) and the mechanical power from cylinder given as follows;

20 $$\begin{aligned} P_y= \frac{1}{T}\int _{0}^{T}(m{\ddot{y}}+c{\dot{y}}+ky) \, \dot{y} \; \text {dt} \end{aligned}$$

For simplicity, the mathematical model of the harvested power is established by assuming the amplitude vibration is sinusoidal for simplicity. As stated by Ding et al.^73^, the sinusoidal wave approximation is reasonably accurate for VIV response. Whereas for galloping, due to its self-induced mechanism, discrepancy is expected. On that basis, the displacement of cylinder in Eq. (17) is derived into velocity and acceleration as follows;

21 $$\begin{aligned} \dot{y}&= y_0 \, \omega \cos (\omega T)\nonumber \\ \ddot{y}&= - y_0 \, \omega ^2 \sin (\omega T) \end{aligned}$$

Taking the previous assumption, the only non-zero energy term is the velocity term^21,37^. Hence, the power produced from the cylinder’s motion in transverse direction of the incoming flow is;

22 $$\begin{aligned} P_y= \frac{c {{\omega }^2y_0}^2}{2} \end{aligned}$$

As reported by Ding et al.^73^, the significant parameters to estimate the harnessable power from the flow-induced vibration are amplitude vibration response and its respective frequency. Previous studies associated with harvesting the energy from flow-induced vibration using electromagnetic transducer came to the same conclusion through their findings from experimental measurements^20,21,38^.

Electromagnetic transducer converts the mechanical power from relative motion of permanent magnet and coil into electricity^78^. For electromagnetic transducer, further investigation on the circuitry load resistance and characteristic of the permanent magnet and coil is necessary^38,79^. Due to its small inertial mass, the power harvested from this transducer is small and limited for micro scale application^78^. The optimization of the harvested power depends on the capability of the transducer. Nevertheless, according to Koide et al.^20^, the damping factor can be assumed equal to the total effective coefficient of magnetism transducer $$c = c_{g}$$. This assumption yields for maximum power generation under the resonance condition with an optimum load resistance of the closed circuit. Note that in this particular study the damping coefficient c is the product of logarithmic damping factor $$\delta$$ and effective mass, m determined by the free damping oscillation test following Maruai et al.^68^. By substituting c = 4$$\pi m \zeta$$$$f_n$$ into the Eq. (22), the harvested power is formulated as below;

23 $$\begin{aligned} P_{harv} = 8{\pi }^3m \zeta {y_{0}}^2 f^2 f_n \end{aligned}$$

The formulated power is used to estimate the power harvested from the flow-induced vibration of the square cylinder throughout this study. Moreover, as suggested by previous study, the efficiency of power conversion is measured through the ratio of harvested power onto the power of fluid. Based on the Bernoulli’s equation, the kinetic energy in the flowing fluid acting onto the cylinder is described by the kinetic pressure as follows;

24 $$\begin{aligned} P_T&= \frac{1}{2} \rho U^2 \end{aligned}$$

where $\rho$ is the fluid density. The pressure generates force which enables the fluid to flow and move the cross section. Therefore, the pressure energy (power) of the flowing fluid over the projected area of cylinder perpendicular to the direction of flowing fluid is calculated by multiplying the kinetic pressure acting unto the cylinder and the flow rate, *Q* of the flowing fluid^20^.

25 $$\begin{aligned} P_{fluid}&= \frac{1}{2} \rho U^2 Q \nonumber \\&= \frac{1}{2} \rho U^2 (UA) \end{aligned}$$

where *A* is the cross section of cylinder. Following Ding et al.^21^, the efficiency of power conversion is calculated as;

26 $$\begin{aligned} \eta&= \frac{P_{harv}}{P_{fluid}}\nonumber \\&= \frac{8{\pi }^3m \zeta {y_{0}}^2 f^2 f_n}{\frac{1}{2} \rho U^3 DL} \end{aligned}$$

where *D* is the side length and *L* is the spanwise length of the cylinder.

## Results and discussion

### Case plate length w/D = 0.5

According to Apelt et al.^50^, placing a splitter plate of length $w/D\leqslant$ 2 in the wake of a circular cylinder organizes the character of flow separation and demonstrates a narrower wake than for a single cylinder. Hypothetically, the alteration of wake flow invokes a different mechanism of motion onto a bluff body. In this section, the influence of flat plate of length *w*/*D* = 0.5 in the wake of square cylinder is investigated. The flat plate is designated downstream to the square cylinder for gap separation ranging between 0.5 $\leqslant G/D \leqslant$ 3.

#### Global properties

**Figure 8:**
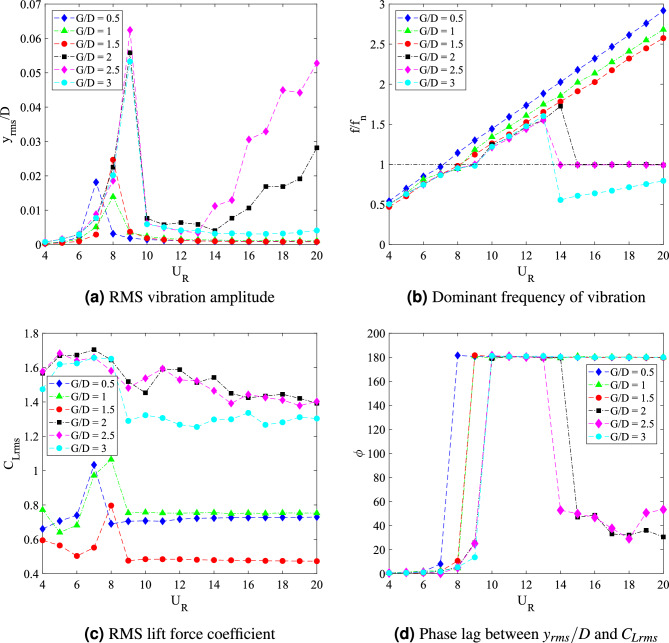
Dynamic response of a square cylinder with flat plate length $w/D =$ 0.5.

In general, varying the downstream flat plate length affects the dynamic response of the square cylinder. The changing of the dynamic response can be grouped according to the gap distances. The dynamic responses associated with the flow-induced vibration of a square cylinder with downstream flat plate length *w*/*D* = 0.5 are illustrated in Fig. 8.

The amplitude response of FIV over a square cylinder with the presence of a flat plate $w/D =$ 0.5 are plotted in Fig. 8a. Common VIV character was observed for 0.5 $\leqslant G/D \leqslant$ 1.5. According to Feng^80^, for a high mass-damping ratio system, there are two branches of VIV character. The initial branch is accompanied by the highest vibration amplitude. Lower branch, on the other hand, yields to the decrement of vibration amplitude. Following that, the coexistence of VIV and galloping was found when the flat plate is further extended at gap separation ranging between 2 $\leqslant G/D \leqslant$ 2.5. The character of FIV within this gap separation range is similar to the case of an isolated cylinder. This finding implied the ineffectiveness of the plate towards the dynamic response of square cylinder for this particular range (2 $\leqslant G/D \leqslant$ 2.5). However, when the flat plate was further extended to G/D = 3, the galloping character was diminished at the higher branch of reduced velocity. The amplitude curve was steadily low indicating the lower branch of VIV and concurrently, suppression of galloping behaviour at $U_R>$ 13. Nonetheless, within the low branch of velocity ranging between 4 $\leqslant U_R \leqslant$ 13 the amplitude response of case *G*/*D* = 3 coincided with the case of *G*/*D* = 2 and *G*/*D* = 2.5 as depicted in Fig. 8a.

**Figure 9:**
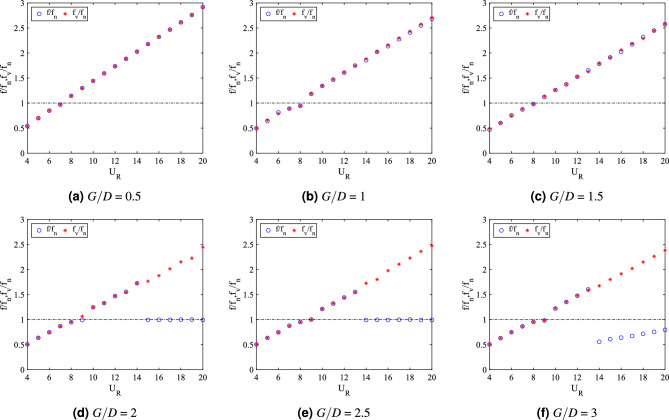
Comparison between dominant frequency of vibration ($f/f_n$) and frequency of vortex shedding ($f_v/f_n$) for plate length *w*/*D* = 0.5.

In addition to the vibration amplitude response, another significant aspect of dynamic response of FIV is the dominant frequency of vibration. For cylinder-plate of length *w*/*D* = 0.5, the dominant frequency of vibration response is presented in Fig. 8b. For cases of 0.5 $\leqslant G/D \leqslant$ 1.5, the frequencies of vibration were linearly increasing with the reduced velocities. VIV lock-in synchronization occurs when $f/f_n$ = 1 at a critical reduced velocity, which was corresponding to the peak of vibration amplitude. Similarly, the vortex shedding frequency is also increasing with the increased of reduced velocity as shown in Fig. 9a–c.

The coinciding trend of both frequency of vibration and vortex shedding frequency reveals the influence of vortex shedding towards the vibration of square cylinder. On the contrary, for cases *G*/*D* = 2 and *G*/*D* = 2.5, the dominant frequency response showed a sharp fall at high reduced velocity region and coincided with the natural frequency of cylinder at $f/f_n$ = 1. Hypothetically, the onset velocity of that breakdown (frequency) is related to the beginning of galloping. The disassociation of vibration character from the influence of vortex shedding is demonstrated in Fig. 9d,e.

For the case of gap separation G/D = 3, the dominant frequency rose with an evident lock-in synchronization associated with the high vibration amplitude at 8 $\leqslant U_R \leqslant$ 9. Towards the higher branch of velocity, a sudden drop of frequency was observed from $U_R$ = 14 suggesting the galloping behaviour. Moreover, as shown in Fig. 9f, the dominant frequency of vibration is diverted from the trend of vortex shedding frequency. This manifests the mechanism of galloping. However, unlike the two previous gap separation cases, the self-induced character of classic galloping ($f/f_n$ = 1) was obscured.

The lift force coefficient which partially contributed towards the flow-induced vibration for cylinder-plate w/D = 0.5 is also presented in Fig. 8c. A significant increment of lift coefficient is observed within the low reduced velocity region $U_R \leqslant$ 9 when the gap separation of cylinder plate was extended. As mentioned previously, for cases $G/D\geqslant$ 2, the character of FIV is associated with the case of an isolated cylinder. This contributed to the rise of lift force coefficient as the effectiveness of passive control is declining. For $G/D \leqslant$ 1.5, the galloping occurrence was successfully eliminated in conformity by the constant lift force coefficient at the lower branch of VIV ($U_R \leqslant$ 9). Although it was apparent that the higher velocity region was corresponding to the VIV behaviour through the low vibration amplitude response, the magnitude of lift coefficient explained contrastingly. The trend of lift force coefficient for case gap separation of G/D = 3 is also similar to the cases of gap separation *G*/*D* = 2 and *G*/*D* = 2.5, of which explained the isolation of vibration response from the vortex shedding influence. Previous deduction of galloping mechanism for this case *G*/*D* = 3 is confirmed through the lift force coefficient trend as shown in Fig. 8c.

The assessment of phase difference between cylinder displacement and lift force signal verifies the difference between VIV and galloping behaviour. Study from Assi et al.^81^ showed that VIV at the initial branch is $\phi$ = 0$^{o}$ and at the lower branch of VIV, $\phi$ = 180$^{o}$. According to Nikitas and Macdonald^76^, for galloping mechanism, the phase lag between displacement and the forcing force was observed around $\phi$ = 60$^{o}$. Thus, it was evident that galloping occurred during higher reduced velocity region as shown in Fig. 8d only for cases 2 $\leqslant G/D \leqslant$ 2.5^76^. Whereas, for other cases including case *G*/*D* = 3, the in-phase lag between the vibration amplitude and lift force was observed during the low reduced velocity region and within the higher reduced velocity region, the out of phase lag was discovered.

**Figure 10:**
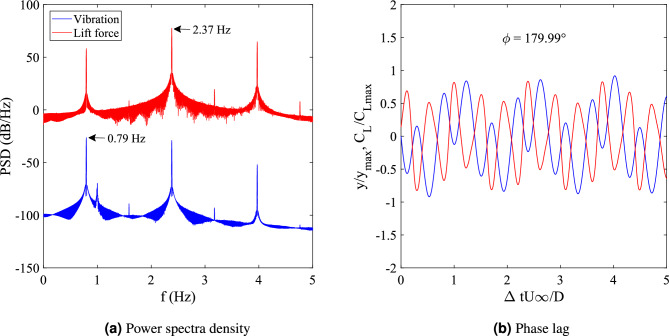
Time series of vibration amplitude (red line) and lift force coefficient with the corresponding power spectrum density and phase lag for case G/D = 3.

The remaining challenge is to define the dynamic regime of case *G*/*D* = 3. Figure 10 encloses the dynamic characteristics of square cylinder when the plate is located at *G*/*D* = 3. The distribution of dominant frequencies for both vibration and lift force coefficient are diverged. The vortex shedding frequency is three times greater than the frequency of vibration as observed in Fig. 10a. The 1:3 synchronization is characterized as a modified galloping by the study from Zhao et al.^82^.

According to Zhao et al.^82^, the dynamic response of such synchronization, although it is due to the galloping occurence, may contradict from the classic case of galloping. This clarifies the peculiar response of low vibration amplitude at high reduced velocity region for case G/D = 3 in Fig. 8a. Another argument sets the ground breaking definition for this case is for a regime to be identified as modified galloping is that the vibration amplitude must be in phase with the lift force fluctuation^82^. This conflict put a halt to this hypothesis as the phase lag found in Fig. 10b is $\phi$ = 179.99$^{o}$ suggesting the out of phase character. According to the dynamic response discussed in this section, there are two regimes of dynamic response for the case of plate length *w*/*D* = 0.5. The first regime was observed when the location of plate is ranging between 0.5 $\leqslant G/D \leqslant$ 1.5 encapsulated of VIV character. Following that, the coexistence of VIV and galloping was well observed for cases of gap separation 2 $\leqslant G/D \leqslant$ 2.5. However, for *G*/*D* = 3, the attribute of dynamic regime is yet to be confirmed due to the conflicting idea of previous study^82^ and the current finding. Hence, for a better explanation of this uncommon feature, flow visualization analysis is demonstrated in this study.

#### Flow visualization

Flow visualization analysis comprises the explanation of flow feature around the square cylinder and confirms the dynamic regime associated with the flow-induced vibration. For a single isolated cylinder, two main vortex patterns were discovered as reported by Maruai et al.^61^. The classic 2S vortex mode found in the initial branch of VIV is characterized by the single vortex shedding in a half cycle downstream of the cylinder. During the lower branch of VIV and galloping, P+S was observed of which demonstrates a single and a pair of vortices per cycle^61^.

**Figure 11:**
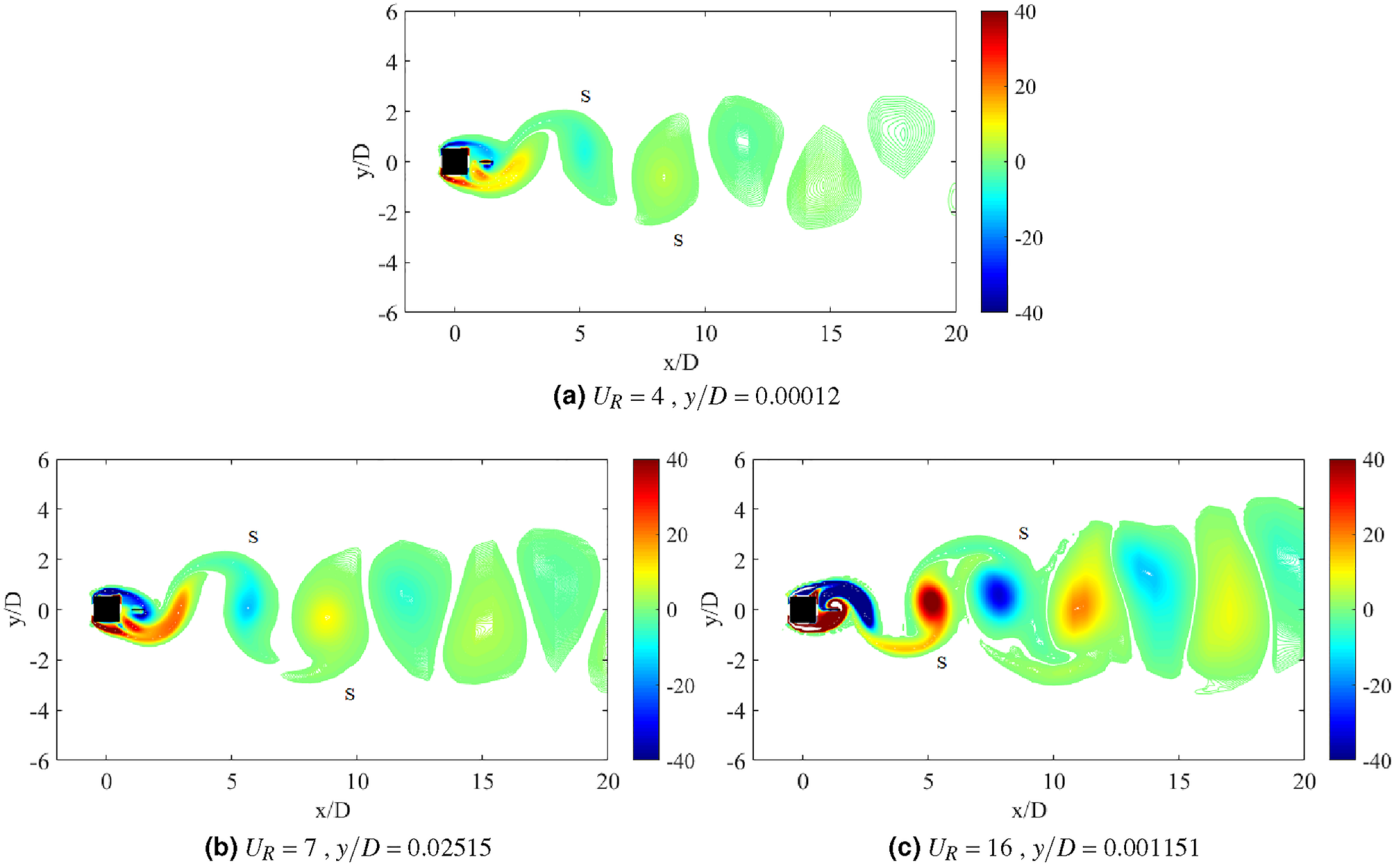
Vortex patterns for flat plate *w*/*D* = 0.5 for *G*/*D* = 0.5.

Through wake modification by placing a flat plate downstream of square cylinder, other vorticity patterns are anticipated with aim to classify the new regimes of flow-induced vibration. Based on the dynamic responses, two regions of FIV were observed when a plate of length *w*/*D* = 0.5 is placed downstream to the square cylinder. The first region was discovered for gap separation 0.5 $\leqslant G/D \leqslant$ 1.5, while the latter was found when flat plate is located at 2 $\leqslant G/D \leqslant$ 2.5. The first region consists of only two branches of VIV, namely, initial and lower. The suppression of galloping for gap separation 0.5 $\leqslant G/D \leqslant$ 1.5 was confirmed by the frequency response and phase lag between the vibration and lift force signals.

Figure 11 shows the vortex pattern at instantaneous time of cylinder’s maximum displacement for gap separation G/D = 0.5. The selection of reduced velocities are corresponding to the initial branch of VIV, maximum peak amplitude of VIV and lower branch of VIV or in some cases, galloping occurrence. The same approach is applied to determine the character of FIV based on the flow visualization analysis for each investigation of different plate length cases and their configuration. In general, the flow over the bluff body creates the downstream vortex street. 2S pattern or two alternating vortices shed in half cycle of oscillation^77^ was observed for $U_R$ = 4,7 and 16. The roll up of vortices from shear layer occurs immediately downstream to the flat plate and increased the vortex formation length. However, the stretching of vortex formation has no effect towards the pattern of vortices for case *G*/*D* = 0.5.

**Figure 12:**
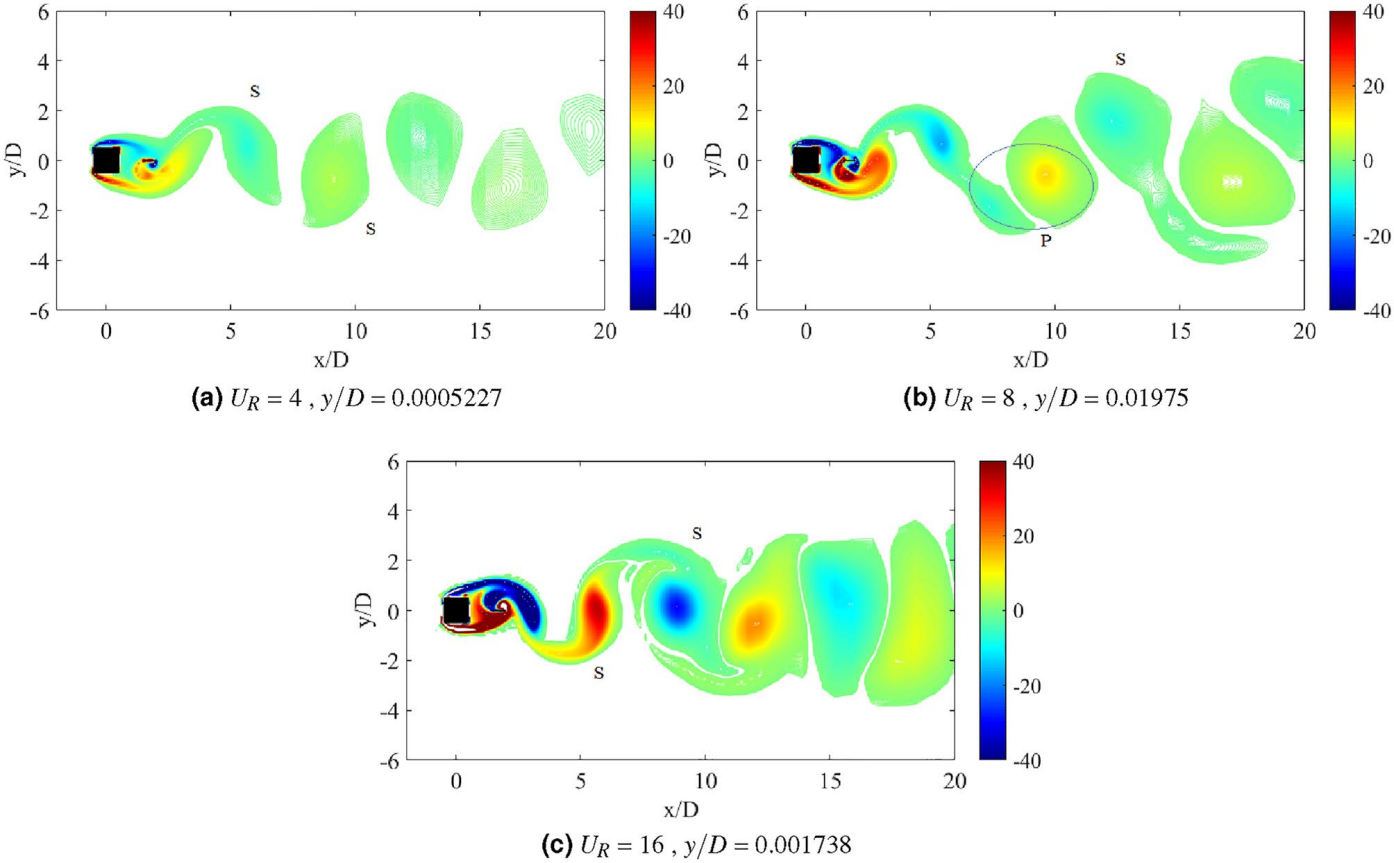
Vortex patterns for flat plate *w*/*D* = 0.5 for *G*/*D* = 1.

Distinct difference of distances between the alternating vortices’ core was observed in Fig. 11. The vortex shedding of higher reduced velocities was relatively more expeditious than the lower reduced velocities due to the decreasing of lateral trajectory between the vortices and the increased of longitudinal distance between vortices^77^. The dynamics of the vortices are similar to the von Karman street vortex for non-vibrating cylinder. This deduces the generation of low magnitude vibration amplitude except for the lock-in synchronization range. Nevertheless, towards expanding the mode change of vortex formation, the example of such character was discovered for the case of *G*/*D* = 1 as depicted in Fig. 12 . Unlike when the gap separation *G*/*D* = 0.5, the vortex formation occurs immediately behind the flat plate. During the initial branch of VIV ($U_R$ = 4), the classic 2S mode is observed. Further increased of reduced velocity ($U_R$ = 8) at lock-in synchronization point, the wake mode changes from 2S to P+S.

The change of modes from vortex street type 2S pattern into a P+S pattern of synchronization is depicted in Fig. 12 as the reduced velocity is increased. This transition from 2S to P+S modes occurs when the critical trajectory wavelength is surpassed due to the increased of imposed reduced velocity^77^. The mechanism of P+S is caused by the asymmetry splitting of alternating vortices by which one of them is sustained its strength to recover. According to Williamson and Roshko^77^, P+S pattern consists of an alternating vortex pair, which shed in the wake and is closely followed by a single vortex. The pairs were formed in curve pathways and returned to the centreline, as the sum of clockwise vorticity in the pair was balanced out with the negative vorticity in the single vortex. Further increased of reduced velocity demonstrates the changes in mode from P+S to 2S. Figure 12c shows that the shear layer convects towards further downstream of the flat plate, hence prevents the asymmetry splitting of vortices. The 2S mode is recovered as illustrated in Fig. 12c at reduced velocity $U_R$ = 16.

**Figure 13:**
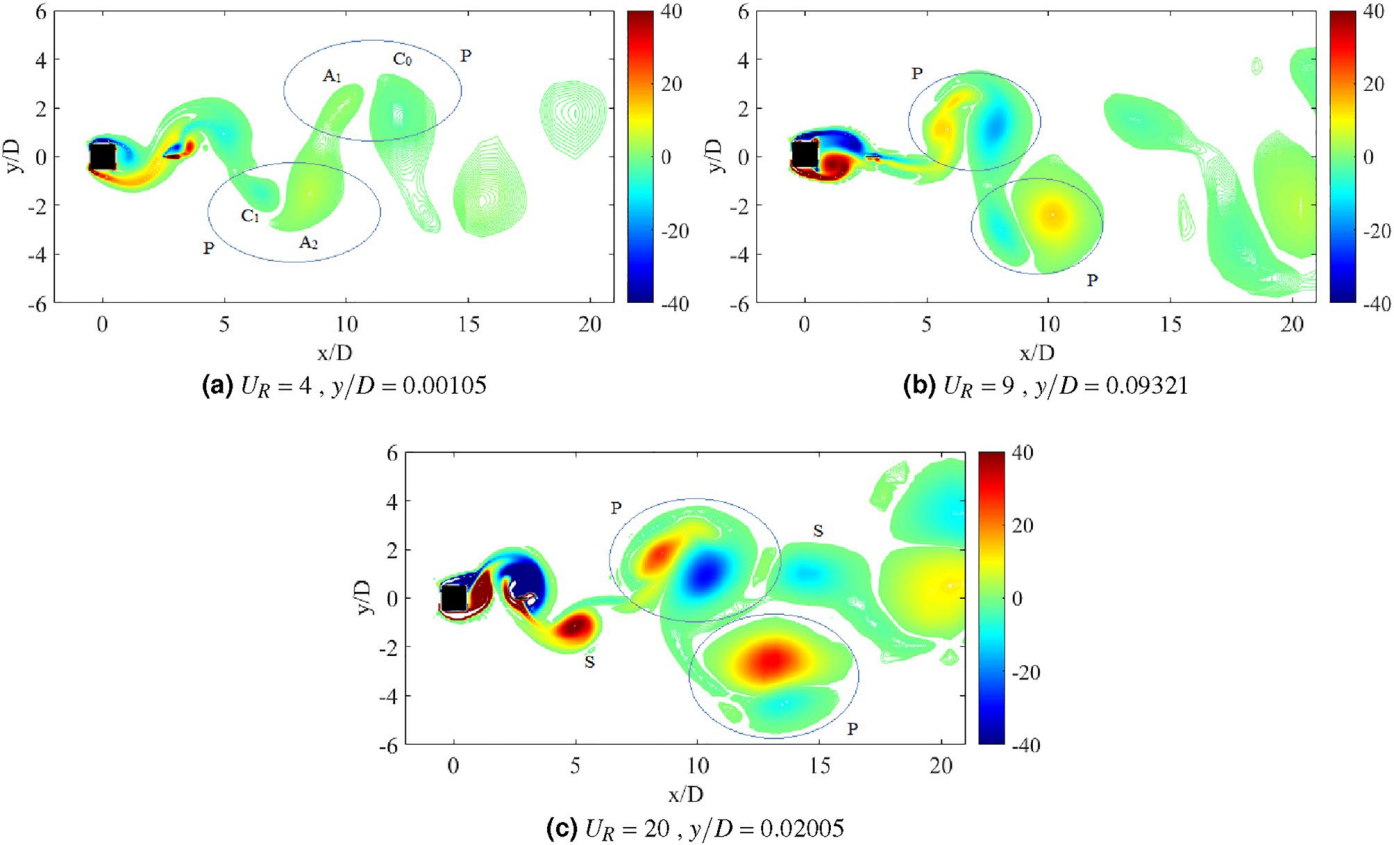
Vortex patterns for flat plate *w*/*D* = 0.5 for *G*/*D* = 2.

The second region consists of the coexistence of VIV and galloping characters, which has similar dynamic response to the isolated square cylinder discussed in Chapter 4. Taking the case of gap separation *G*/*D* = 2 as an example, the vortex patterns formed when plate of length *w*/*D* = 0.5 are illustrated in Fig. 13. In contrast to the first region, the vortex pair was completely formed inside the gap between the cylinder and flat plate. The transition of 2S mode into 2P mode was observed at $U_R$ = 4. Further increased of velocity to $U_R$ = 9, an established 2P vortex pattern is discovered during the maximum amplitude response of VIV. While at $U_R$ = 20, which galloping behaviour was predicted, the S+2P+S pattern was discovered. The inconsistency of shedding vortices (frequency of vortex shedding) from the frequency of vibration indicates the ineffectiveness of vortex shedding as the driving mechanism of cylinder’s motion^83^.

**Figure 14:**
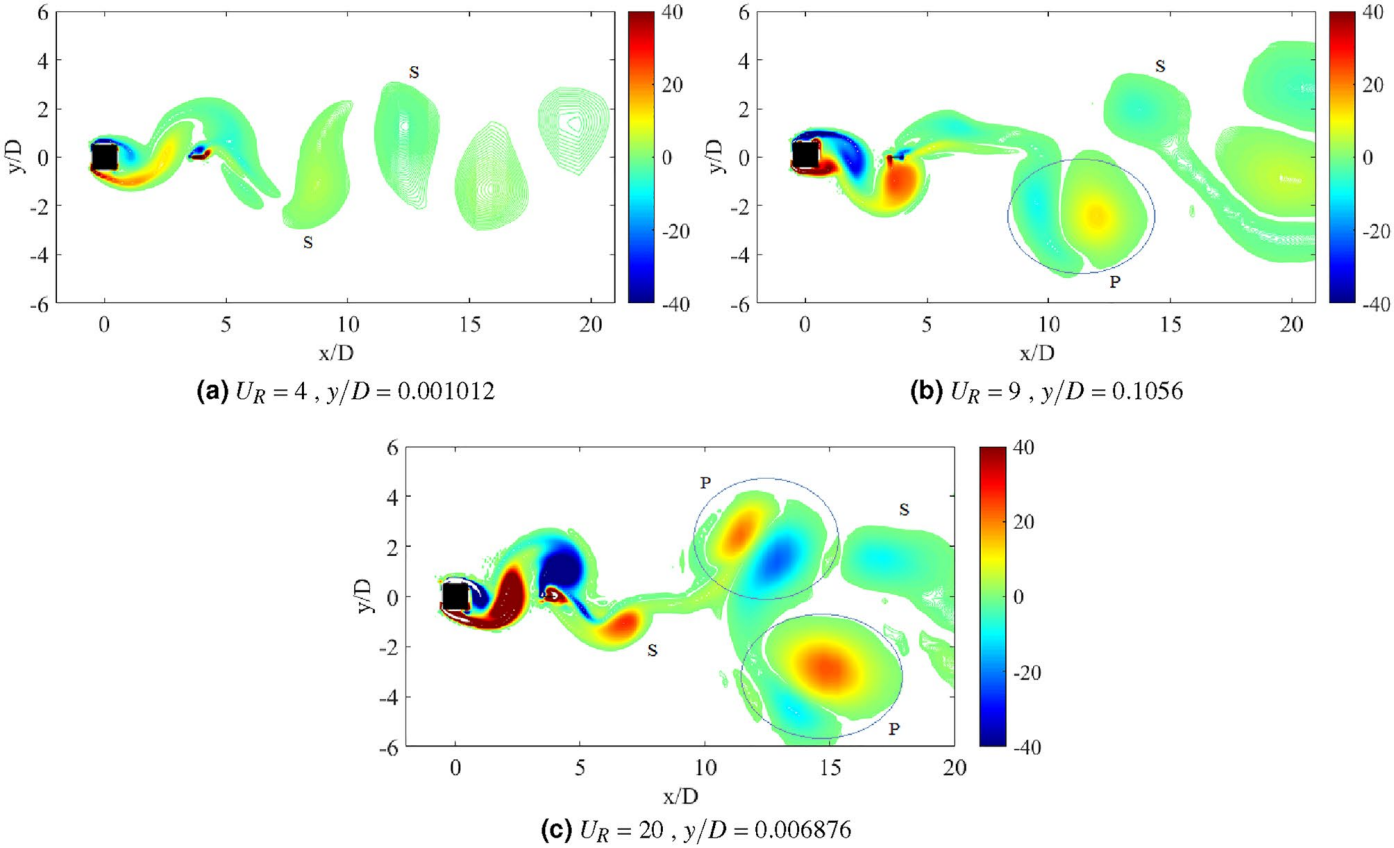
Vortex patterns for flat plate *w*/*D* = 0.5 for *G*/*D* = 3.

The vortex patterns formed in the wake when the flat plate was located at *G*/*D* = 3 are presented in Fig. 14. During the initial branch of VIV, 2S vortex pattern was apparent downstream of the plate. Whereas, as the reduced velocity is increased to $U_R$ = 9 when the peak of highest vibration amplitude is exhibited, an evident P+S pattern was observed. Based on the dynamic responses presented in Fig. 8, there were a few conflicting issues regarding the case with *G*/*D* = 3. In general, galloping is characterized by the increasing high amplitude. Contrarily, the amplitude response of *G*/*D* = 3 at a higher branch of velocity was relatively very low and thus, evoked the inclusion of *G*/*D* = 3 in the second region. Nonetheless, based on the vortex pattern in Fig. 14c, the S+2P+S pattern was discovered. This pattern was comparable to the vortex mode found in Fig. 13c, which identifies the galloping mechanism. Thus, for a gap separation at *G*/*D* = 3, a flat plate length of *w*/*D* = 3 has established the modified galloping occurrence towards the square cylinder.

### Case plate length w/D = 1

Flat plate with length of *w*/*D* = 1 is considered as the medium plate length throughout this study. For a rigid square cylinder, two flow regimes were identified based on the flow structure analysis when a plate length *w*/*D* = 1 is present. The first regime lies when the gap separation between the cylinder and plate is $G/D \leqslant$ 2. For this regime, the completed vortex formation appears downstream of the plate. Whereas, the second regime is identified by the complete vortex formation inside the gap separation between cylinder and flat plate. This regime was observed for cases 2.5 $\leqslant G/D \leqslant$ 3. Meanwhile, a transition regime was found around gap separation 2 $\leqslant G/D \leqslant$ 2.5. A transition of flow regime was found by Ali et al.^46^ at a critical gap $G_c/D$ = 2.3 when a plate of length of *w*/*D* = 1 located downstream to a square cylinder. Similar cylinder-plate configuration is opted in this study for a non rigid cylinder.

#### Global properties

**Figure 15:**
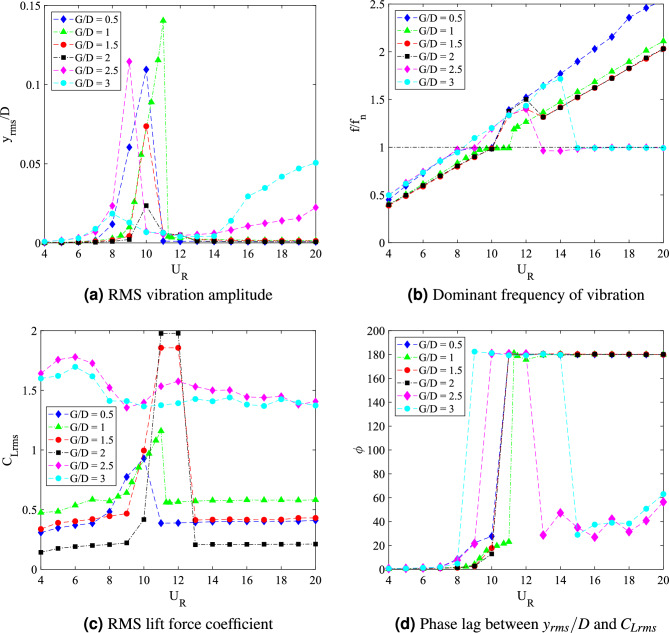
Dynamic response of a square cylinder with flat plate length *w*/*D* = 1.

The dynamic response for cylinder-plate of *w*/*D* = 1 is presented in Fig. 15. The root-mean-square (rms) vibration amplitude of FIV can be seen in Fig. 15a as a function of the reduced velocity. An increasing pattern of vibration amplitude was observed before a sudden fall around $U_R$ $\approx$ 11 for cases 0.5 $\leqslant G/D \leqslant$ 2. The highest peak vibration amplitude is discovered when flat plate is located *G*/*D* = 1 from the cylinder at reduced velocity $U_R$ = 11. However, with the further increased of gap separation (1.5 $\leqslant G/D \leqslant$ 2), the peak amplitude decreases. For most cases, a significant low vibration amplitude was found during the higher branch of velocity (11 $\leqslant U_{R}$ $\leqslant$ 20). Meanwhile, for cases 2.5 $\leqslant G/D \leqslant$ 3, there is an apparent rise of amplitude starting at $U_R$ $\approx$ 14 indicating the galloping character. Based on the vibration amplitude plot, there are two regimes of dynamic response. The first regime was observed for gap separation 0.5 $\leqslant G/D \leqslant$ 2 and the second regime encapsulated cases of gap separation 2.5 $\leqslant G/D \leqslant$ 3.

**Figure 16:**
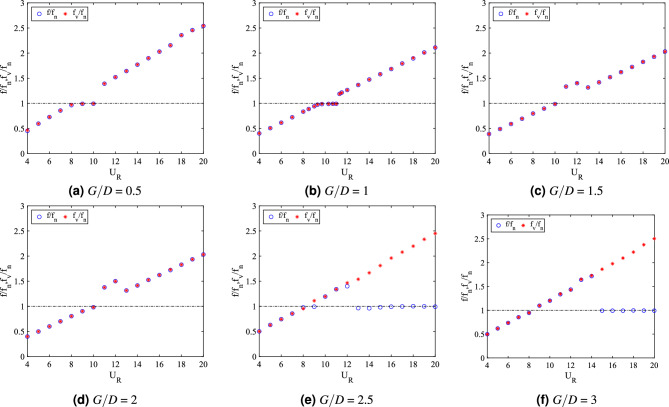
Comparison between dominant frequency of vibration ($f/f_n$) and frequency of vortex shedding ($f_v/f_n$) for plate length *w*/*D* = 1.

The plots for dominant frequency of vibration are presented in Fig. 15b. For the case *G*/*D* = 0.5, the trend of frequency plot concurs with the pure VIV character associated with initial branch of VIV ($U_R$ = 4-8), lock-in synchronization ($U_R$ = 9-10) and lower branch of VIV ($U_R$ = 11-20). The same trend of frequency response was found for case *G*/*D* = 1. Additionally, in correspondence to the increasing vibration amplitude for case *G*/*D* = 1 within range of reduced velocities 9 $\leqslant U_R \leqslant$ 11, the dominant frequency of vibration coincides with the natural frequency manifesting the lock-in synchronization of VIV as presented in Fig. 16b. This case in particular has the most potential for energy harvesting for low reduced velocity. Generally, a good trade off of high amplitude at low reduced velocity is expected when gap separation between cylinder and flat plate is $G/D\leqslant$ 1.

The frequency plot in Fig. 16c,d for cases of gap separation between 1.5 $\leqslant G/D \leqslant$ 2 has depicted an abrupt jump of frequency of vibration at $U_R$ = 11-12 and followed by the drop of frequency at $U_R$ = 13-20. Similar trend was also observed for the vortex shedding frequency in Fig. 16c,d. The matching trends of the frequency of vibration and vortex shedding frequency reveals the character of VIV. For $G/D\geqslant$ 2.5, the lower branch of velocity demonstrated the character of VIV when the frequency trend is similar to the previous cases of different gaps separation. However, towards the higher branch of velocity, the drop of frequency plot suggested the coexistence of two different mechanisms. One of them is corresponding to the motion of cylinder and the other is due to the vortex shedding. This describes the self-induced character of galloping. The collapsed of frequency of vibration into the natural frequency suggests the dissociation of vibration behaviour from the influence of vortex shedding. Therefore, the distinctive trend of frequency of vibration and the shedding frequency in Fig. 16e,f is related to the galloping mechanism in the higher branch of reduced velocity.

According to the variation of lift force coefficient over reduced velocities plotted in Fig. 15c, two distinctive regimes can be identified. For cases of gap separation between 0.5 $\leqslant G/D \leqslant$ 1, similar pattern of response as the vibration amplitude was revealed. However, with the extension of gap separation between 1.5 $\leqslant G/D \leqslant$ 2, the lift force coefficient was relatively lower than those found for cases 0.5 $\leqslant G/D \leqslant$ 1 particularly at the initial branch and lower branch of VIV. This concurs with the low vibration amplitude experienced by the cylinder when the plate is located in vicinity of gap separation between 1.5 $\leqslant G/D \leqslant$ 2. During the VIV lock-in synchronization (11 $\leqslant U_R\leqslant$ 12), an aggressive jump of lift force coefficient was demonstrated. The character of lift force during this intermittent period is consistent with the relatively lower peak of amplitude response and the sudden jump of frequency of vibration. Nevertheless, only VIV behaviour was observed in this regime and thus, the effect of flat plate is apparent and significant.

The second regime, in contrast to the first regime, exhibits the galloping occurence at high velocity region. This is similar to the case of isolated cylinder. The effect of flat plate in this regime becomes weaker with the extension of gap separation. It is possible to correlate this finding to the previously published data by Ali et al.^46^, which reports two regimes of flow discovered for splitter plate of 1D. In correspondence to the report by Ali et al.^46^, the critical gap for the case of plate length *w*/*D* = 1 is assumed to occupy the transition gaps 2 $< G/D<$ 2.5. The transition gap in this study is also expected to lie within the gap separation 2 $<G/D<$ 2.5, which is between the first and second regime.

The division of dynamic regimes for plate length *w*/*D* = 1 is confirmed through the phase lag analysis depicted in Fig. 15d. Regime 1 (0.5 $< G/D<$ 2) encapsulates of VIV character for all reduced velocities. The in phase response between fluctuating lift and vibration is observed during lower branch of velocity. Also, the out of phase ($\phi$ = 180$^o$) behaviour is discovered at higher branch of velocity. Regime 2 encloses the coexistence of VIV and galloping throughout 4 $\leqslant U_R \leqslant$ 20. The in phase and out of phase characters were evident at low reduced velocities, which respectively represented the initial and lower branch of VIV. Galloping was well observed at high reduced velocities when the phase lag abruptly fell from $\phi$ = 180$^o$ to around $\phi$ = 60$^o$^76^ which occurs at reduced velocity when the vibration amplitude increases as shown in Fig. 15a. The starting of galloping for Regime 2 is also confirmed through the comparison between the dominant frequency response and the vortex shedding frequency in Fig. 16e,f.

### Flow visualization

**Figure 17:**
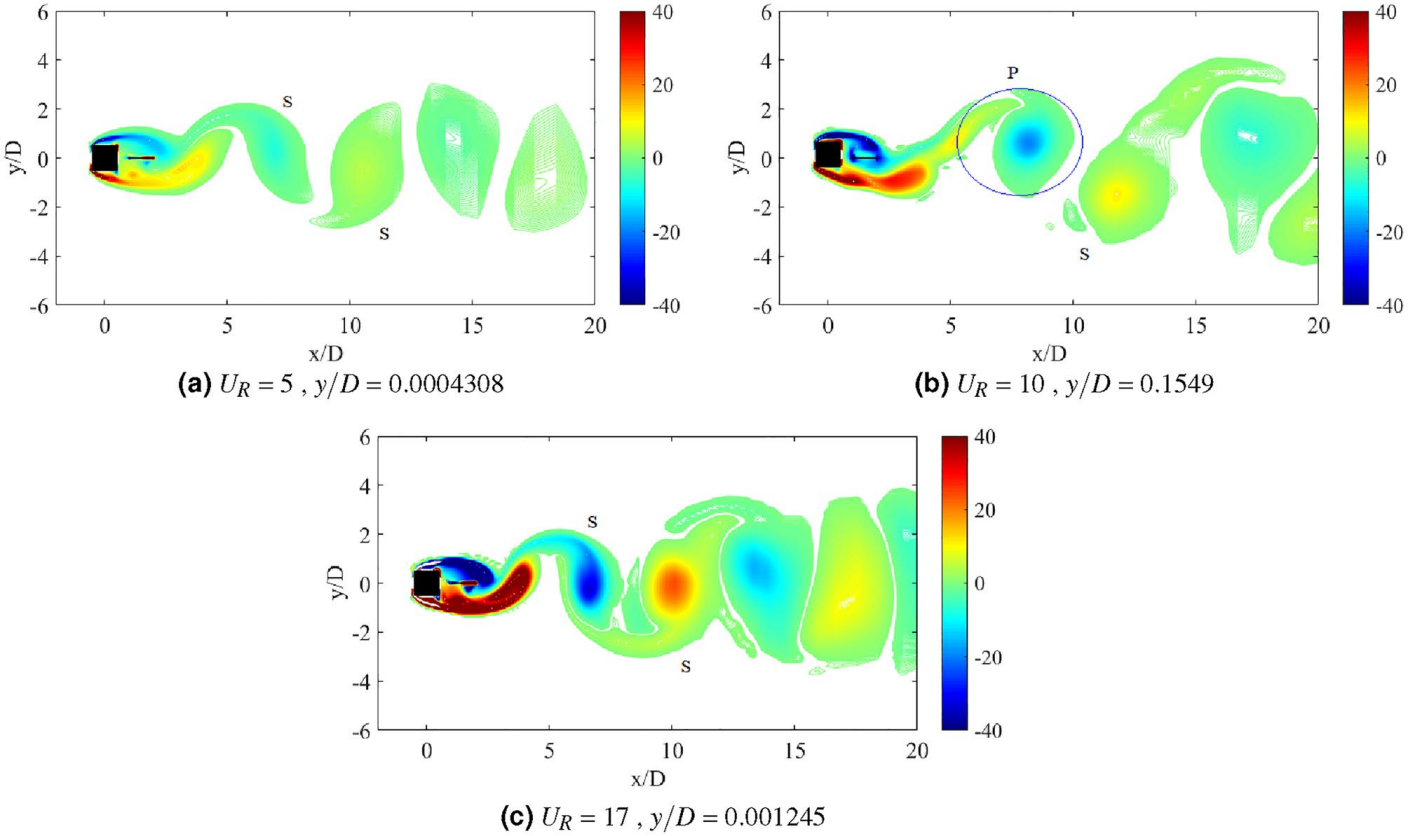
Vortex patterns for flat plate *w*/*D* = 1 for *G*/*D* = 0.5.

The dynamic response of a medium plate in Fig. 15 has predetermined two dynamic regimes for cases of gap separation between 0.5 $\leqslant G/D \leqslant$ 3. The first regime is encapsulated by cases of gap separation between 0.5 $\leqslant G/D \leqslant$ 2. The vortex pattern in regards to this regime is plotted in Fig. 17 for case *G*/*D* = 0.5. 2S vortex pattern was found during the $U_R$ = 5 and $U_R$ = 17, while at $U_R$ = 10 when the vibration amplitude is maximum, P+S vortex mode was observed. The asymmetry P+S mode comprises a single and a pair of vortices shed during a half cycle of vibration. The total amount of vorticity of the upper pair P is equal to the vorticity in the single S. The clockwise vortex of the pair (blue) is more tenacious than the anticlockwise vortex and thus, maintained the pair to convect within the wake centreline^77^.

**Figure 18:**
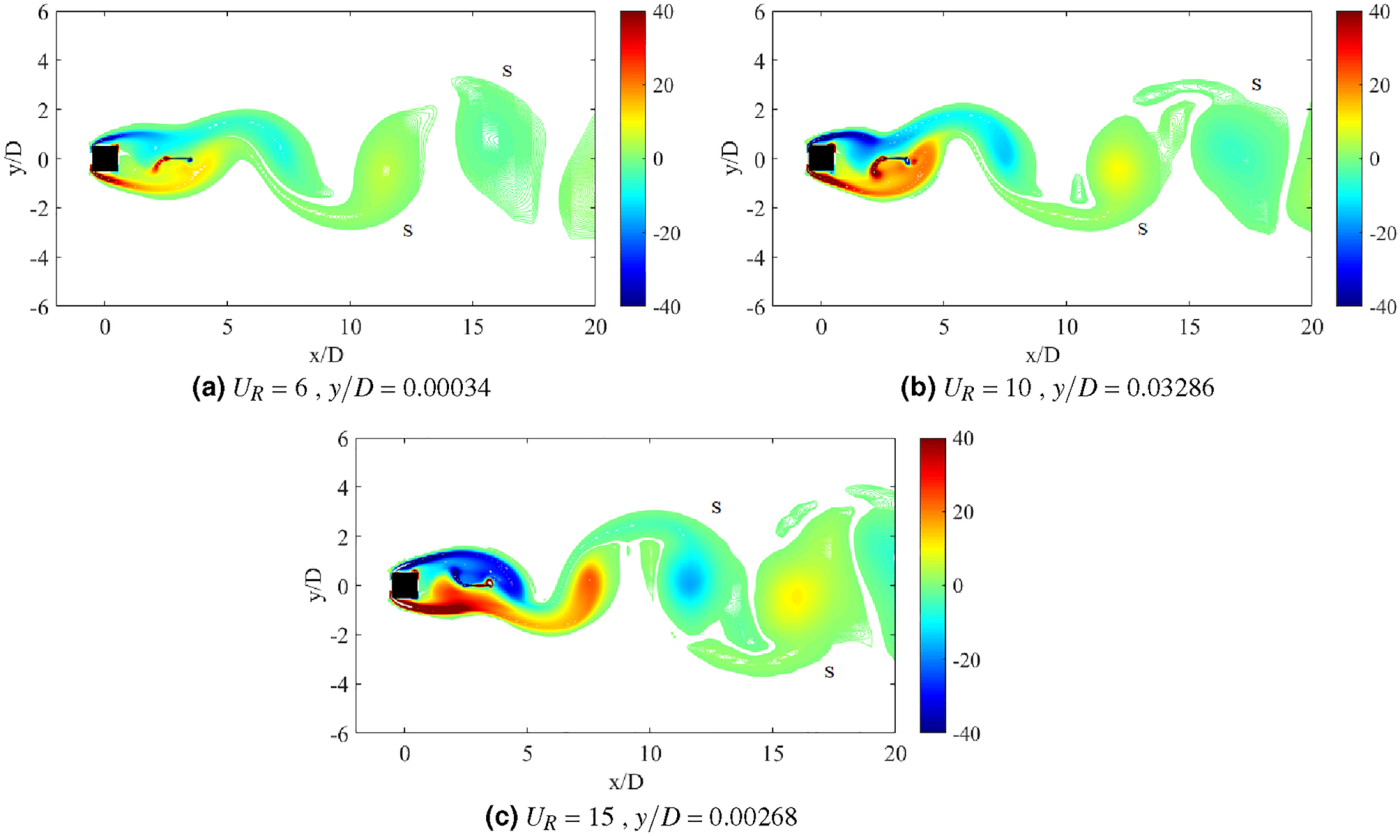
Vortex patterns for flat plate *w*/*D* = 1 for *G*/*D* = 2.

However, as the gap separation is further extended to *G*/*D* = 2, the P+S vortex mode has returned into 2S vortex mode at the highest peak of vibration amplitude ($U_R$ = 10) as presented in Fig. 18. 2S vortex pattern was consistently observed throughout the considered range of reduced velocities in this case (*G*/*D* = 2). The suppression of galloping at the higher branch of reduced velocity was evident in this regime as the patterns of vortex shedding are consistently corresponding to the driving mechanism of cylinder’s motion based on the dominant frequency plot as shown in Fig. 16.

**Figure 19:**
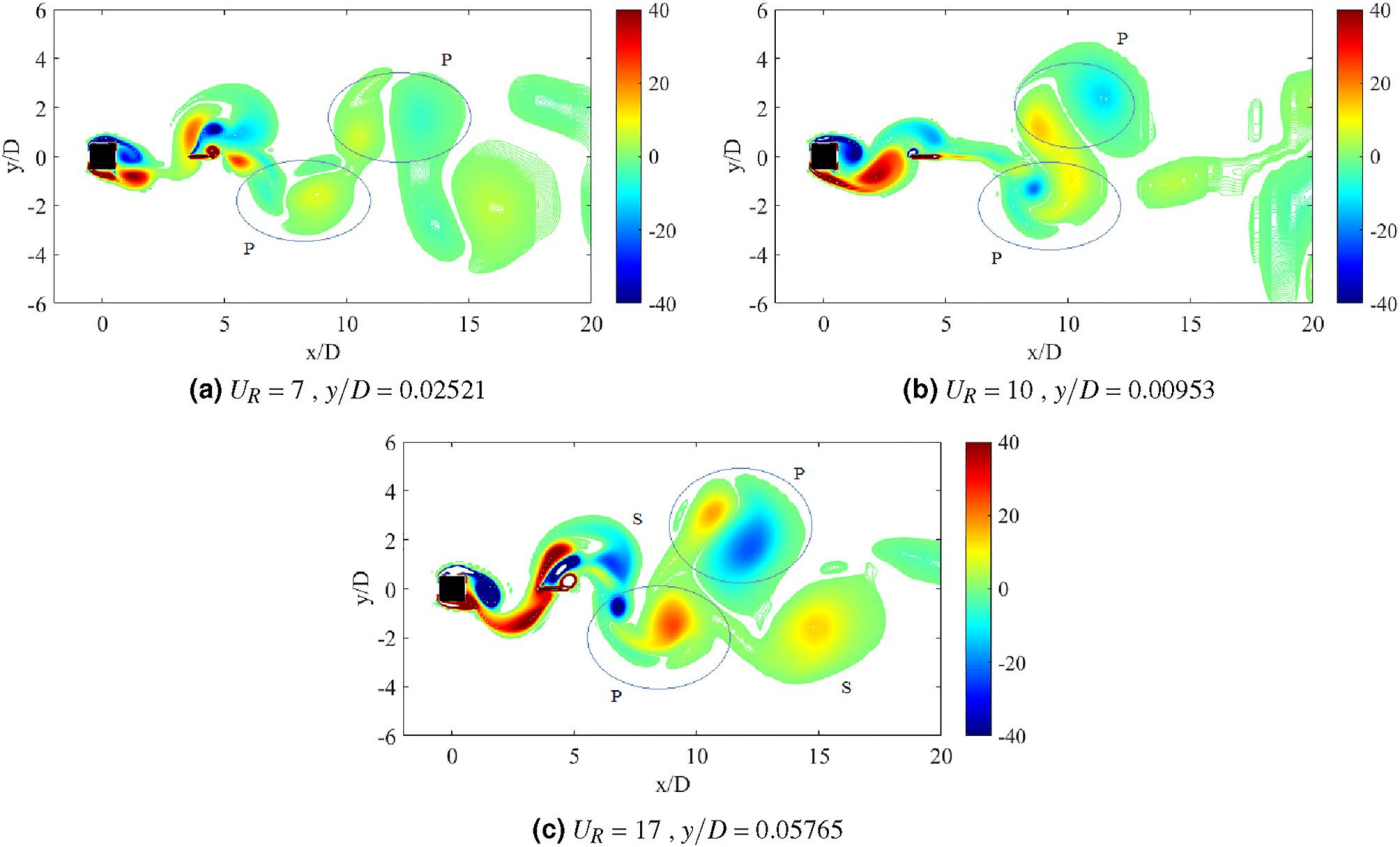
Vortex patterns for flat plate *w*/*D* = 1 for *G*/*D* = 3.

Regime 2 encloses cases of gap separation between 2.5 $\leqslant G/D \leqslant$ 3. In this regime, the combination character of VIV and galloping was discovered based on the dynamic response analysis. A fully developed vortex formation was consistently observed inside the gap separation *G*/*D* = 3 for all reduced velocities as can be seen in Fig. 19. The transition of 2S mode $\rightarrow$ 2P mode was perceived in the initial branch of VIV as depicted in Fig. 19a for $U_R$ = 7. As the reduced velocity increased, a well developed 2P vortex mode was revealed in Fig. 19b reveals the vortex mode of 2P. This vortex mode is displayed in the lower branch of VIV at $U_R$ = 10. Figure 19c presents the vortex pattern found in the galloping region. S+2P+S vortex mode was observed at $U_R$ = 20. Similar pattern suggesting the ineffectiveness of vortex influence towards the motion of square cylinder was also discovered from the small plate case. Therefore, the importance of the plate in controlling wake flow is revoked here.

### Case plate length w/D = 3

Apelt and West^51^ found for a normal flat plate (sharp edge face) splitter plate of length 0 $\leqslant L/D \leqslant$ 3 in the wake influences the vortex shedding formation. According to previous study, for a long plate $L/D>$ 3, the suppression of vortex shedding is potent. Therefore, to observe the effects of long plate towards the dynamic of square cylinder, a flat plate of length *w*/*D* = 3 is considered for inspection in this study.

#### Global properties

**Figure 20:**
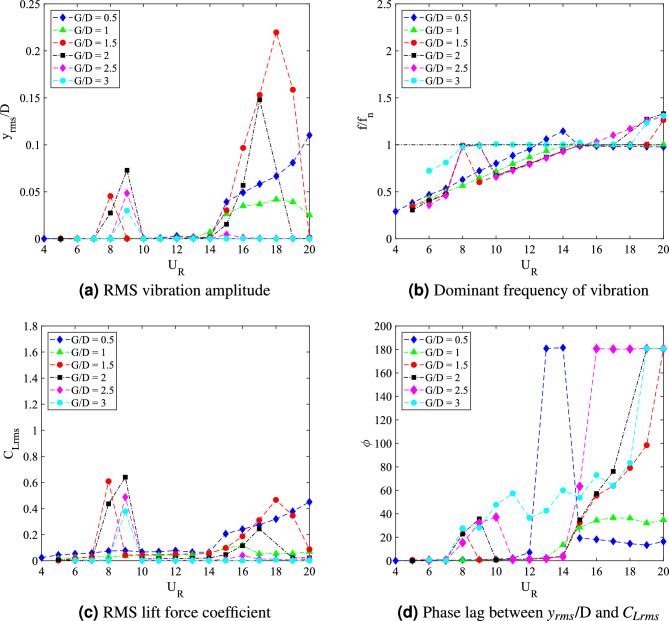
Dynamic response of a square cylinder with flat plate length *w*/*D* = 3.

The dynamic response for case of plate length *w*/*D* = 3 is disclosed in Fig. 20. In general, the dynamic response for this long plate has a significant dissimilarity with the small and medium plate length. The most remarkable difference was observed within the higher branch of velocity region of which the second peak was discovered. In the vicinity of higher velocity region, the vibration amplitude increased impressively for $G/D<$ 3. Nonetheless, the common VIV peak occurred at the lower branch of velocity for $G/D>$ 1.5. This peak of VIV at low velocity region was also found for cases of plate length *w*/*D* = 0.5 and *w*/*D* = 1.

From the vibration amplitude plot in Fig. 20a, three dynamic regimes were identified. The first regime is characterized by the increasing vibration amplitude within high branch of reduced velocity. For cases 0.5 $\leqslant G/D \leqslant$ 1, no apparent lock-in synchronization character was observed at the lower branch of reduced velocity. However, the vibration amplitude has significantly increased starting from 15 $\leqslant U_{R} \leqslant$ 20 for case *G*/*D* = 0.5. Rather than the increasing trend of vibration amplitude as suggested from the case of *G*/*D* = 0.5, the vibration amplitude decreases from 18 $\leqslant U_R \leqslant$ 20 when the plate is extended to *G*/*D* = 1. As the gap separation is further increased, a peculiar vibration amplitude response was discovered. The two peaks of vibration amplitude were found when the plate is located within the gap separation range of 1.5 $\leqslant G/D \leqslant$ 2.5. The first peak developed in proximity of low branch of reduced velocity of which the lock-in of VIV is commonly expected. Meanwhile, the second peak was observed during the high branch of reduced velocity. The second peak exhibits relatively higher vibration magnitude than the first peak. The third regime comprises relatively low vibration amplitude throughout the considered velocities as the gap separation between cylinder and flat plate increased to *G*/*D* = 3. The suppression of vibration amplitude was observed in the vicinity of high branch of reduced velocity.

**Figure 21:**
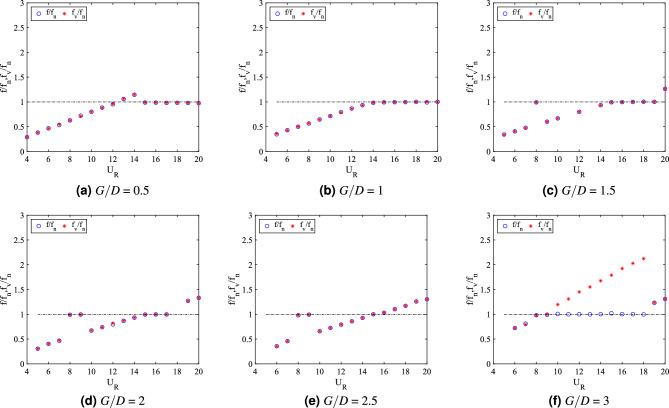
Comparison between dominant frequency of vibration ($f/f_{n}$) and frequency of vortex shedding ($f_{v}/f_{n}$) for plate length *w*/*D* = 3.

Previously, the increasing vibration amplitude within the higher branch of velocity was associated with the galloping phenomenon. However, for this particular plate length (*w*/*D* = 3), the comparison between the dominant frequency of vibration and vortex shedding frequency in Fig. 21a,b has demonstrated otherwise. The pattern of dominant frequency of vibration concurs with the vortex shedding frequency suggesting that the vibration is highly influenced by the vortex shedding mechanism. This deduces that the increasing vibration amplitude for cases *G*/*D* = 0.5 and *G*/*D* = 1 can be classified as vortex-induced vibration. The establishment of two peaks of VIV in Regime 2 is confirmed through the frequency plot and fluctuating force coefficient plot in Fig. 20b,c. The lock-in character prevails during the low and high region of velocity. For a better resolution of such character, the comparison between dominant frequency of vibration and vortex shedding frequency is plotted in Fig. 21c–e. This character is profoundly identical to the VIV group II as reported by Shiraishi and Matsumoto^84^. According to them, the coexistence of leading and trailing vortices separation influences this particular type of VIV. The main reason of such character is due to the slenderness of the bluff body^84^. In this case, the cylinder-plate configuration is mimicking the slender bluff body and entrains the shear layers to move further downstream.

Meanwhile, as the gap separation between cylinder and flat plate is increased to *G*/*D* = 3, the amplitude response is very low. As depicted in Fig. 20c, the lift force is remarkably small as compared to other cases, which explains the finding by Apelt and West^51^. For a relatively long plate case ( $L/D>$ 3), the vortex shedding is suppressed, thus mitigating the vibration amplitude. However, Fig. 21d reveals the separation of driving mechanism between the vibration and vortex shedding. According to Zhao et al.^82^, this phenomenon can be described as modified galloping. The collapsed of frequency of vibration into the natural frequency in Fig. 21f clarifies the character of modified galloping for case G/D = 3. Although the vibration amplitude found within the interval of 10 $\leqslant U_{R} \leqslant$ 18 was low, the dependency of vibration towards the driving mechanism of vortex shedding is withdrawn. Besides, the phase lag between the vibration and vortex shedding presented in Fig. 20d have shown a contradicting remark of the lower branch of VIV in which the low vibration amplitude was initially expected. Referring to previous study, the trend of phase lag plot observed for case of G/D = 3 is revealed around $\phi$ $\approx$ 60$^o$ and related to the galloping mechanism^76^. Thus, in the third regime, galloping phenomenon was demonstrated during reduced velocity 10 $\leqslant U_{R} \leqslant$ 18.

#### Flow visualization

**Figure 22:**
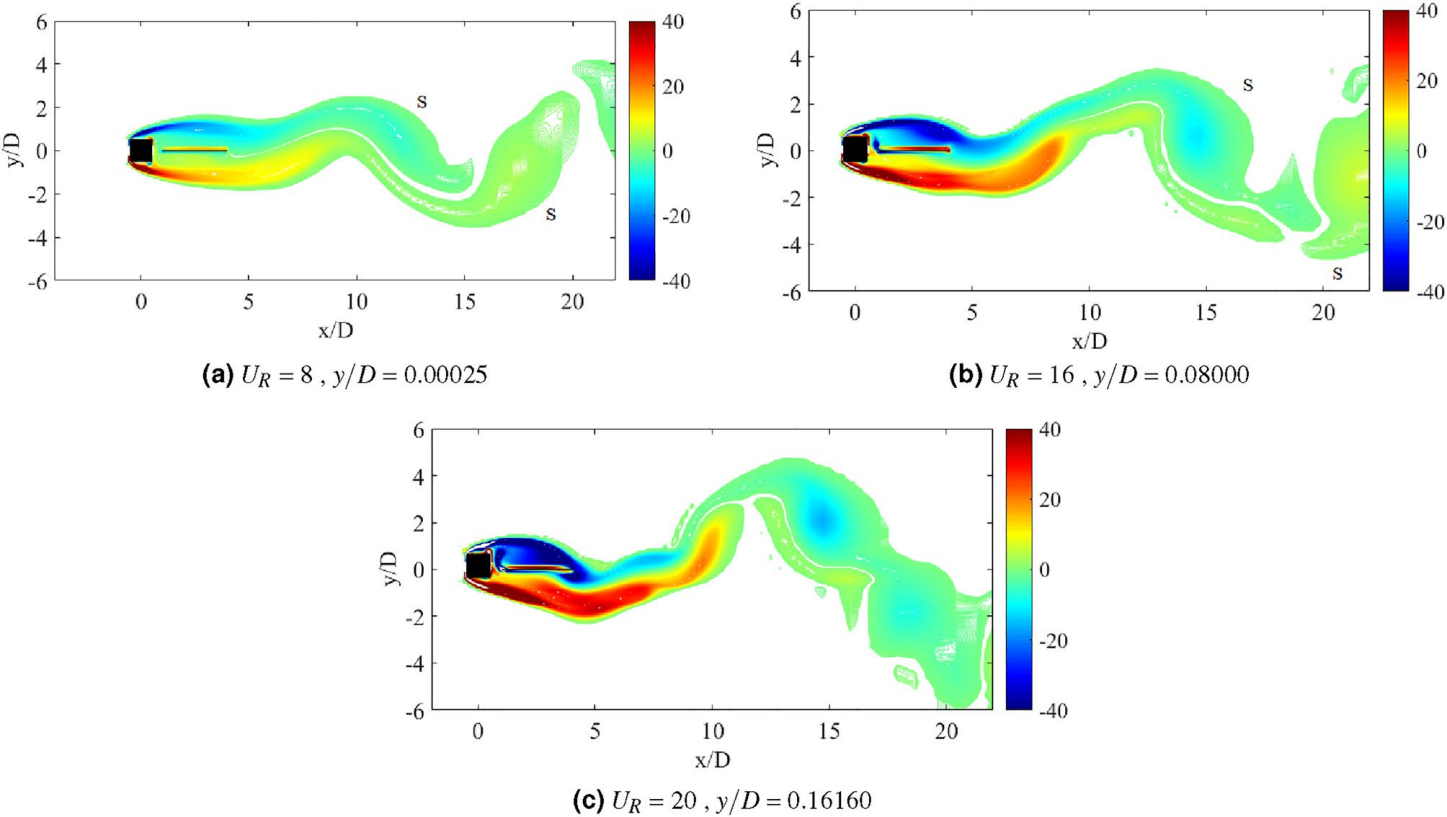
Vortex patterns for flat plate *w*/*D* = 3 for *G*/*D* = 0.5.

Based on the dynamic response disclosed for a square cylinder with long plate (*w*/*D* = 3) at different gap separation, three regimes were identified. The first regime encloses the case of gap separation in the range 0.5 $\leqslant L/D \leqslant$ 1. In general, by using a long plate, the vortex formation is delayed further downstream of the plate. For a small gap distance *G*/*D* = 0.5, the vortex patterns are illustrated in Fig. 22.

**Figure 23:**
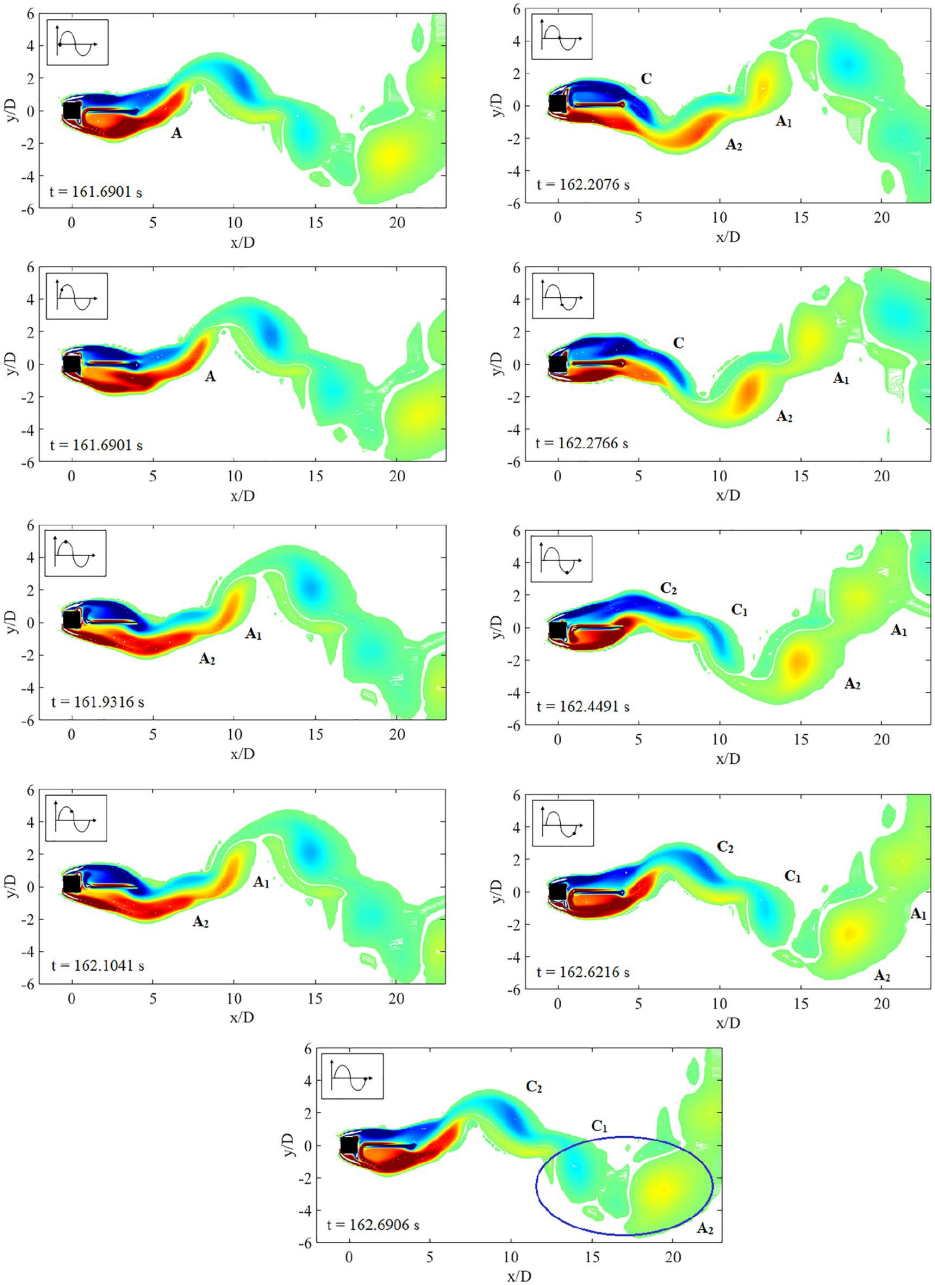
The formation of 2P vortex in the wake of a long plate at $U_{R}$ = 20 for 1 cycle of oscillation (**C**$_{1,2}$—clockwise vortices and **A**$_{1,2}$ - anti-clockwise vortices).

2S pattern was consistently found during $U_{R}$ = 8 and $U_{R}$ = 16. The transition mode from 2S $\rightarrow$ 2P was observed during $U_{R}$ = 16. As the reduced velocity increases, the trajectory wavelength is also increased resulting an early roll up of shear layer behind flat plate as shown in Fig. 22b. The formation of a second vortex (of the same direction as initial vortex) was observed for $U_{R}$ = 20 in Fig. 22c. This pair of vortices is classified as 2P vortex mode^77^. The formation 2P mode for this case is illustrated in Fig. 23.

According to Wlliamson and Roshko^77^, the transition of 2S mode into the 2P mode is due to the increase in trajectory wavelength. The formation of 2P mode is feasible when the timing of vortex formation is shorten by half of cycle and thus, the convection of individual vortex into two like-signed vortices is accelerated. Based on Fig. 23, the vortex to observe is A, which reaches the rear of flat plate comparatively early than the other two cases of different plate length due to the increased of reduced velocity and trajectory wavelength. Vortex **A** was divided into **A**$_1$ and **A**$_2$ due to the acceleration phase of trailing vortices. When the cylinder located at y/D = 0, Vortex **C** is formed. The cycle of acceleration causes a pair of trailing vortices **C**$_1$ and **C**$_2$ to repeat. The two-like signed vortices C$_1$ and C$_2$ convected away and vortex **C**$_1$ were pairing up with vortex **A**$_2$ resulting in a P mode. Herein, for a cycle of oscillation two pairs of vortices were formed and called 2P vortex mode.

**Figure 24:**
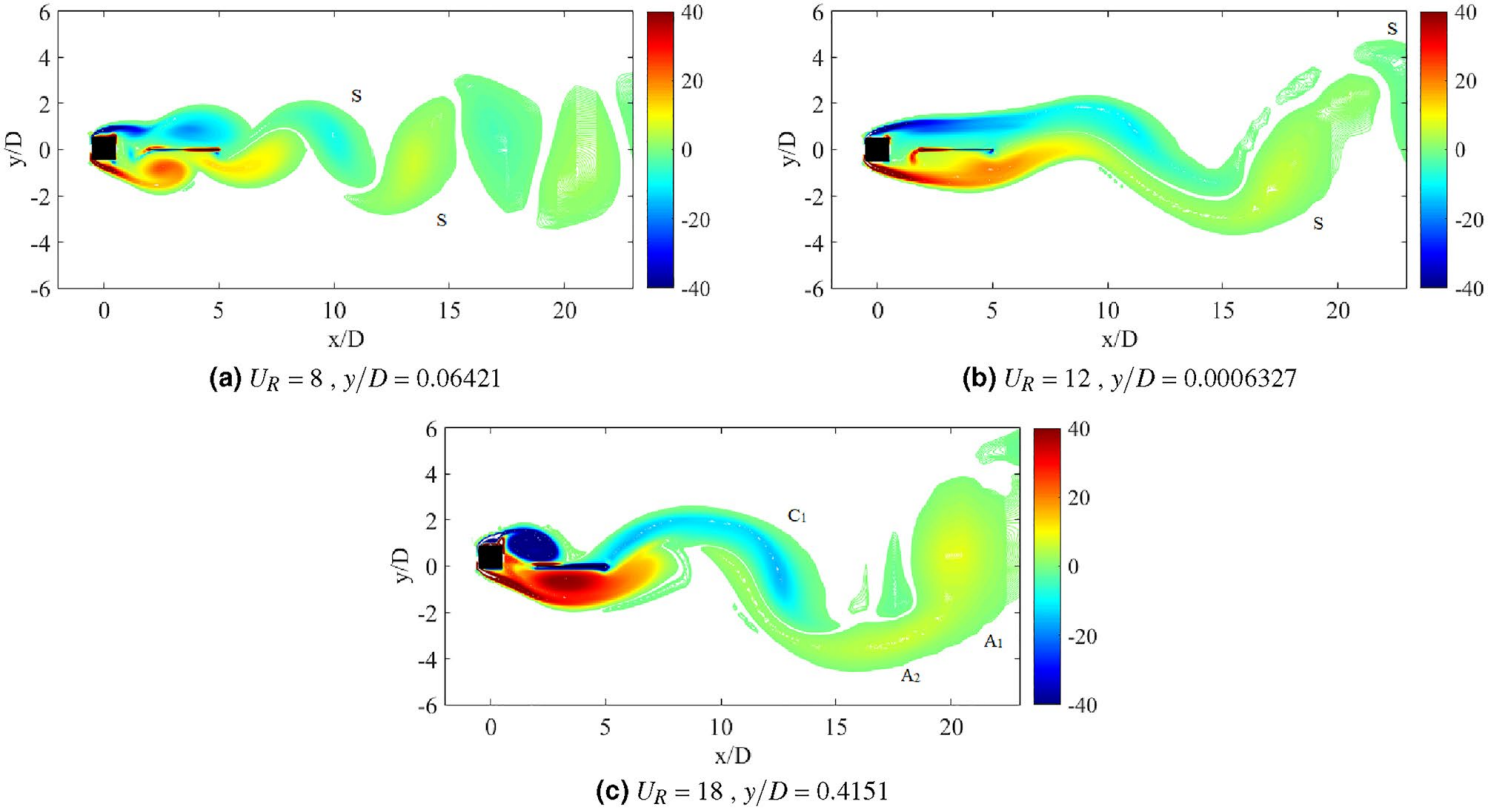
Vortex patterns for flat plate *w*/*D* = 3 for *G*/*D* = 1.5.

The formation of vortices also occurred at the rear of flat plate in Regime 2 as presented in Fig. 24. A 2S vortex pattern was found when the distance of cylinder and plate is *G*/*D* = 1.5 at lower branch of velocity in Fig. 24a. The 2S mode of resonant synchronization was obtained at$U_{R}$ = 8 correlates with the first peak of amplitude observed in Fig. 20a. The plate inhibits the saddle point of vortices and thus disperse the vortices into 2S pattern despite the elongated formation length. However, as the velocity is increased, the vortex formation is delayed by the flat plate. The vortices were formed further downstream of the plate and significantly suppressed the vibration due to the low vortex strength. This explains the desynchronization of vibration and vortex shedding mechanism from their natural state found in Fig. 21c. The behaviour of vortex formation for *G*/*D* = 0.5 in Fig. 22c is repeated for *G*/*D* = 1.5 at high velocity region. As stated previously, the resemblance of a slender body character found in previous study by Shiraishi and Matsumoto^84^ is depicted in Fig. 24. Herein, the 2P mode of vortex pattern is obtained when the plate of length *w*/*D* = 3 located at *G*/*D* = 1.5 behind the flat plate.

**Figure 25:**
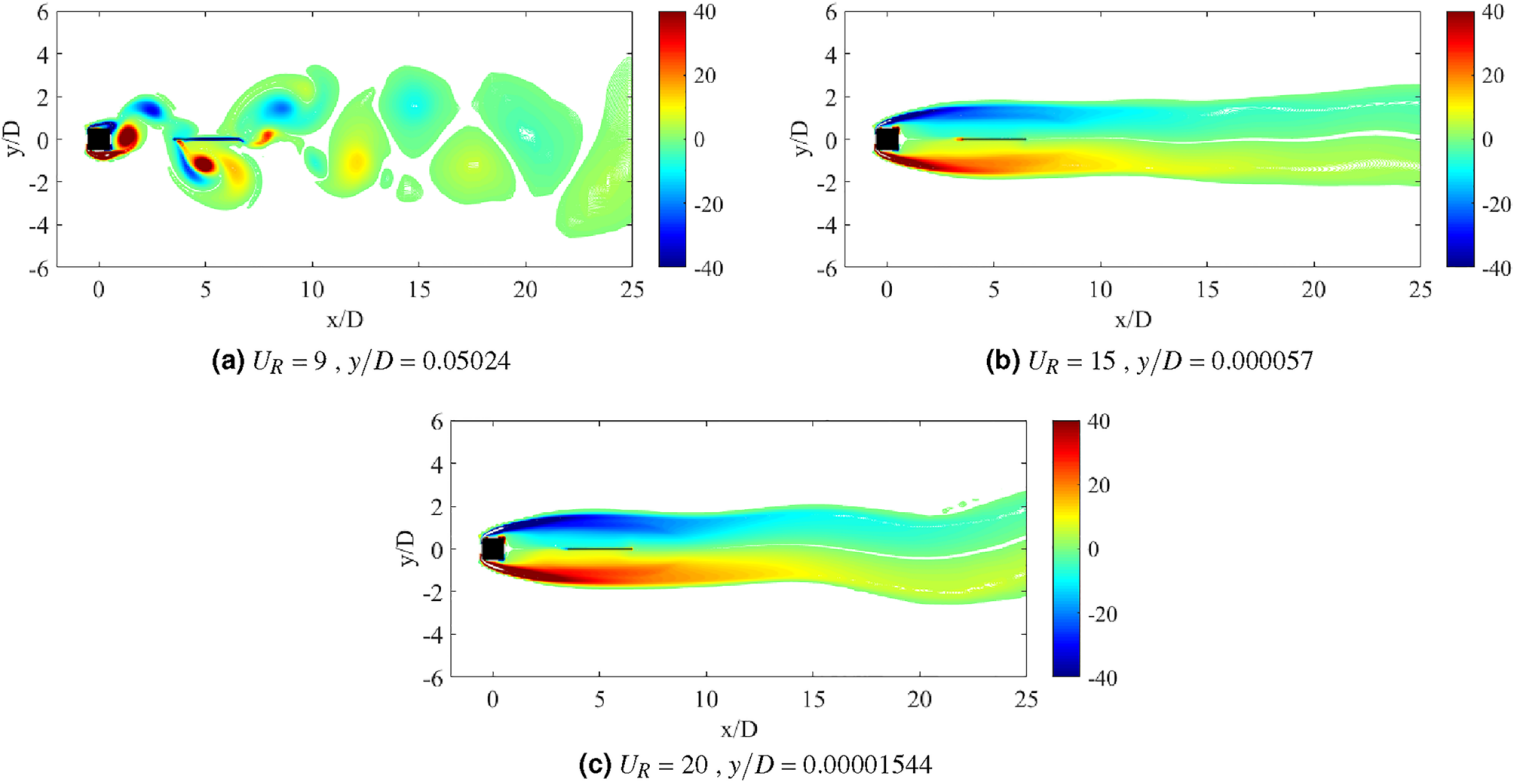
Vortex patterns for flat plate *w*/*D* = 3 for *G*/*D* = 3.

A fully developed vortex formation was observed inside the vicinity of gap separation when the flat plate is located at *G*/*D* = 3 during $U_{R}$ = 9 as shown in Fig. 25. Following that, an alternating 2S vortex mode prevailed at the rear of flat plate. However, as the reduced velocity is increased to $U_{R}$ = 15, no vortex pattern was found neither behind the cylinder nor the flat plate. The further increased in reduced velocity to $U_{R}$ = 18 shows no recovery in vortex pattern.

**Figure 26:**
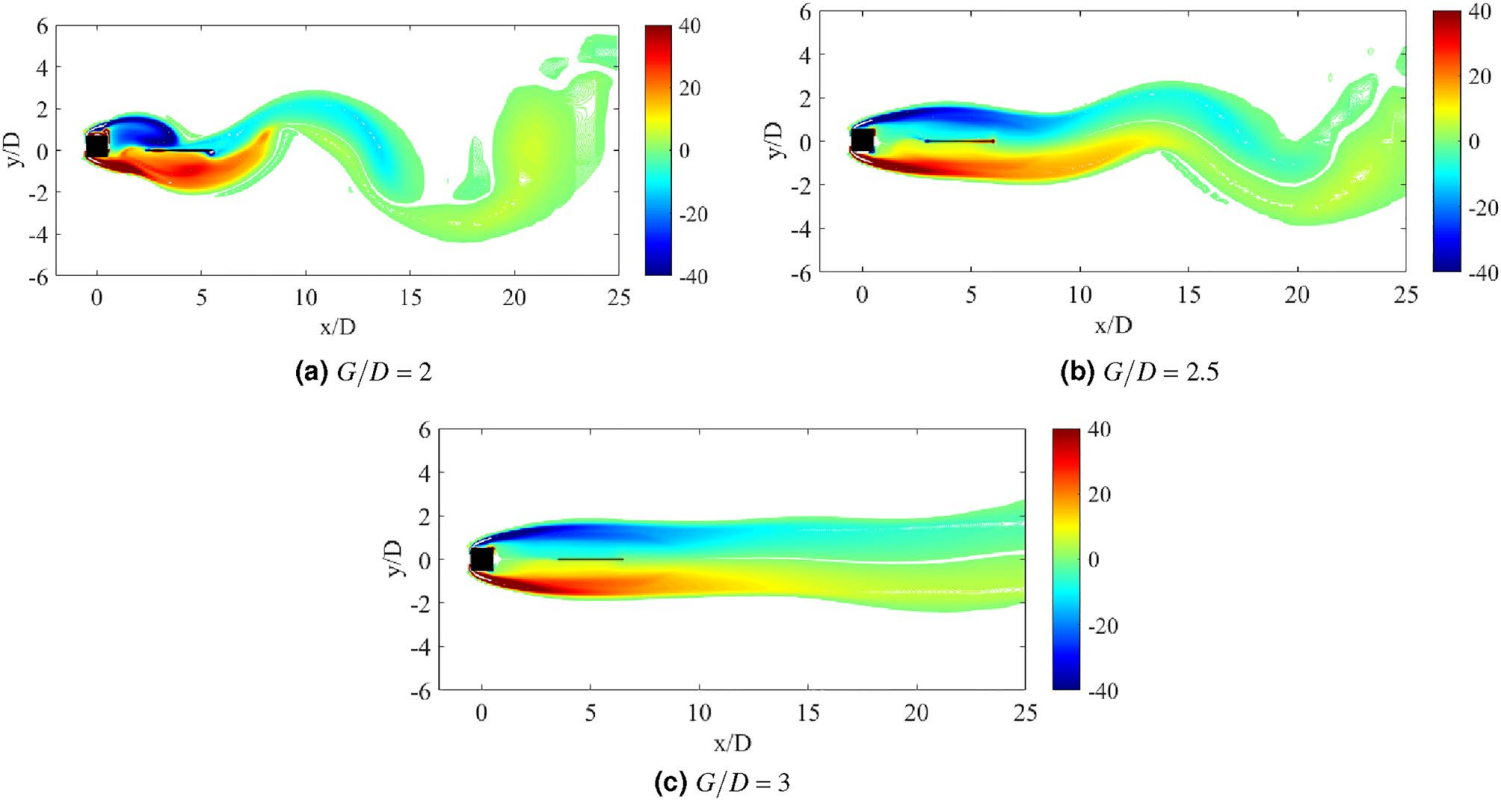
Instantaneous vorticity pattern of flow past a square cylinder with a downstream flat plate (*w*/*D* = 3) at $U_{R}$ = 18.

In general, the shear layers are further recirculating with the increased of reduced velocity. Based on Fig. 26, the extension of shear layers recirculation also occurs due to the elongated flat plate length. Under the flat plate control, the recirculation is suppressed and the flow moves along further downstream of the flat plate. Therefore, the vortex shedding occurrence ceases towards the large gap separation *G*/*D* = 2.5 and *G*/*D* = 3. The absence of vortex shedding is due to the growth of vortex formation length when the *G*/*D* is increased. The growth of vortex formation length is associated with the decrease of base suction and two-dimensional Reynolds stress^49,85^.

### Harvested power estimation

The harvested power estimation for all cases concerning the interaction of cylinder-plate configuration is based on the harvested power equation. The credibility of harvested power equation was verified through the comparison of magnitude and pattern response with the measured power from previous study^68^. In the same context, the harvested power was also substantially influenced by the vibration amplitude and its frequency. Furthermore, the power conversion efficiency is also discussed in this section to determine the most relevant and most adequate flat plate length as a vibration enhancer for energy harvesting application. The results of calculated harvested power and its efficiency for each cylinder-plate configurations with variation of plate length *w*/*D* = 0.5, 1 and 3, are presented respectively below.

**Figure 27:**
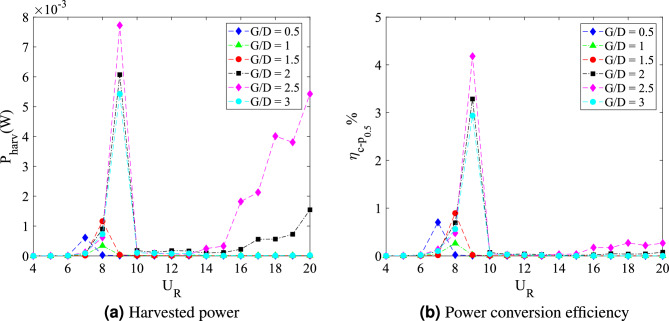
Harvested power estimation and power conversion efficiency for flat plate of length *w*/*D* = 0.5.

The harvested power and its efficiency for cylinder-plate configuration with *G*/*D* = 0.5 in Fig. 27. In general, the harvested power trend is correlated to the trend of vibration amplitude response. The power conversion efficiency, however, is also influenced by the incoming reduced velocity in addition to the vibration amplitude. The degree of effectiveness due to the reduced velocity is significant as the limitation of wind speed is crucial in this study. For the case of flat plate length *w*/*D* = 0.5, infinitesimally small harvested power was observed throughout the considered reduced velocity range when the gap separation was $G/D \leqslant$ 2. The delays of the lock synchronization, which corresponds to the highest peak of energy harvested, were discovered with increasing gap separation. In this first regime ($G/D \leqslant$ 2), the peak of harvested power was obtained for reduced velocity ranging between 7 $\leqslant U_{R} \leqslant$ 9.

In the second regime, a significant hike of power was discovered during low branch velocity, particularly at $U_{R}$ = 9 when the gap separation of cylinder and plate was extended to $G/D \geqslant$ 2 and followed by a sudden drop of power magnitude. The highest harvested power obtained in this regime was experienced by the square cylinder when the flat plate is located at *G*/*D* = 2.5 with $P_{harv}$ = 7.72 x 10$^{-3}$ W. Further increased in reduced velocity revealed the increment of harvested power starting from reduced velocity $U_{R}\geqslant$ 15 for cases of gap separation *G*/*D* = 2 and *G*/*D* = 2.5. The growth of harvested power within the higher reduced velocity range is due to the galloping occurrence. Nonetheless, as the gap separation is further extended to *G*/*D* = 3, the harvested power was eliminated given that the vibration amplitude obtained for this particular case was also suppressed.

According to the power conversion efficiency depicted in Fig. 27b, it is evident that the percentage of efficiency is comparatively higher within the proximity of lock-in synchronization for VIV as to the galloping occurrence. The efficiency of power conversion increases with the increase of gap separation ranging between 1 $\leqslant G/D \leqslant$ 2.5. The most efficient gap separation for this case (*w*/*D* = 0.5) is *G*/*D* = 2.5 with efficiency percentage of 4.29 $\%$. As the second regime for the cylinder-plate of length *w*/*D* = 0.5 manifested the character of vibration trend for an isolated cylinder, it can be deduced that the significance of plate length *w*/*D* = 0.5 is negligible for the energy harvesting application. Moreover, as shown in Fig. 27a, the harvested power for flat plate length *w*/*D* = 0.5 was also comparatively lower than the cases of plate length *w*/*D* = 1 and *w*/*D* = 3. The summary of maximum harvested power and the efficiency for cylinder-plate of length *w*/*D* = 0.5 is listed in Table 7.

**Table 7:** Summary of maximum harvested power and its efficiency for plate length *w*/*D* = 0.5 with different gap separation.

Gap separation	Reduced velocity	Wind velocity	Harvested power	Fluid power	Efficiency
*G*/*D*	$U_{R_{max}}$	*U*	$P_{harv_{max}}$	$P_{fluid}$	$\eta _{\hbox {c-p}_{0.5}}\%$
0.5	7	2.6	0.00061	0.08	0.76
1	8	2.9	0.00034	0.12	0.28
1.5	8	2.9	0.00116	0.12	0.96
2	9	3.3	0.00607	0.18	3.37
2.5	9	3.3	0.00772	0.18	4.29
3	9	3.3	0.00543	0.18	3.01

**Figure 28:**
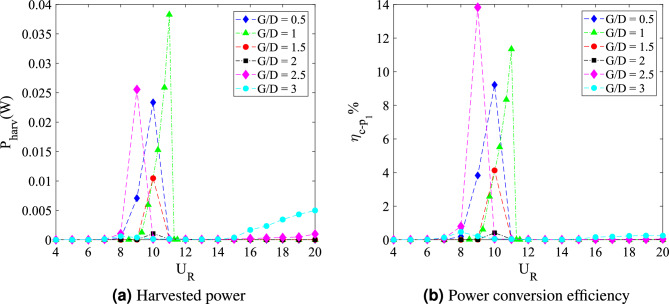
Harvested power estimation and power conversion efficiency for flat plate of length *w*/*D* = 1.

The harvested power and efficiency of power conversion for configuration cylinder-plate of *w*/*D* = 1 are displayed in Fig. 28. As previously mentioned in Sect. "Case plate length w/D = 1", two regimes of FIV were disclosed. For the first regime, in which the interaction of plate towards the wake of cylinder is impressive, the increased of harvested power is obtained within the vicinity of reduced velocity ranging between 10 $\leqslant U_{R}\leqslant$ 11. Following the highest peak of power magnitude was the abrupt decrement of power, which is due to the desynchronization between the vortex shedding and vibration response. It is noteworthy that in this regime, the galloping occurrence is fully mitigated which explains the suppression of the harvested power in the higher branch velocity. The highest generated power of $P_{harv}$ = 3.827 $\times 10^{-2}$ W in this regime revealed when the gap separation was *G*/*D* = 1 and at reduced velocity $U_{R}$ = 11. This particular case (*G*/*D* = 1) demonstrated a broader range of lock-in synchronization, which simultaneously produced an exceptional higher harvested power and thus, could expand the working velocity range for the harvester.

The combined character of VIV and galloping exhibited in the second regime represents the recovery of an isolated square cylinder character. An impressive high harvested power prevailed when the plate is located at *G*/*D* = 2.5 from the cylinder. The maximum harvested power obtained in this case was $P_{harv}$ = 2.555 $\times 10^{-2}$ W. However, as the gap separation was further extended to *G*/*D* = 3, a remarkable fall of harvested power was observed in the range of lock-synchronization. Towards the higher branch of velocity starting from $U_{R}$ = 15, the harvested power gradually increased indicating the recovery of galloping character and the elimination of plate interference towards the square cylinder behaviour.

Based on the efficiency plot in Fig. 28b, there were two outstanding cases that bolstered the credibility of flat plate w/D = 1 to enhance the harvested power. Case of gap separation G/D = 1 represents the first regime with the efficiency of power conversion $\eta _{\hbox {c-p}_{1}}$ = 11.29 $\%$ in reference to the power of fluid. Whereas, from the second regime, the most efficient gap separation was disclosed at *G*/*D* = 2.5 with the efficiency percentage of 14.19. This impressive efficiency degree led to a more comprehensive and further investigation of this plate length. The remaining maximum harvested power and its efficiency are respectively listed in Table 8 according to the cases of variation gap separation.

**Table 8:** Summary of maximum harvested power and its efficiency for plate length *w*/*D* = 1 with different gap separation.

Gap separation	Reduced velocity	Wind velocity	Harvested power	Fluid power	Efficiency
*G*/*D*	$U_{R_{max}}$	*U*	$P_{harv_{max}}$	$P_{fluid}$	$\eta _{\hbox {c-p}_{1}}\%$
0.5	10	3.7	0.02335	0.25	9.34
1	11	4	0.03827	0.32	11.29
1.5	10	3.7	0.01046	0.25	4.18
2	10	3.7	0.00105	0.25	0.42
2.5	9	3.3	0.02555	0.18	14.19
3	20	7.4	0.00499	1.99	0.25

**Figure 29:**
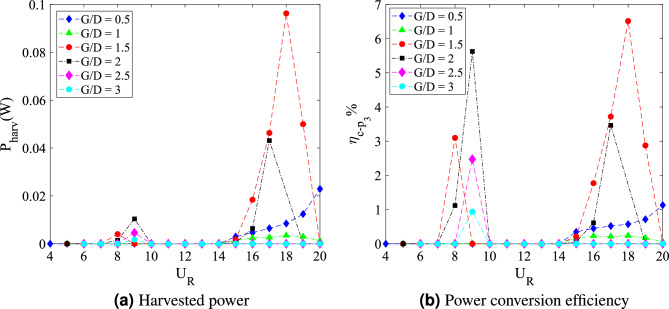
Harvested power estimation and power conversion efficiency for flat plate of length *w*/*D* = 3.

Three regimes of FIV were determined in Case Plate Length W/D = 3 section when a flat plate of length *w*/*D* = 3 is used. For this particular case, the harvested power and power conversion efficiency are presented in Fig. 29. The first regime demonstrated an increasing trend of harvested power for reduced velocity $U_{R} \geqslant$ 15 as shown in Fig. 29a. The domination of higher branch of velocity to harvest significantly high magnitude of power continued in the second regime when the gap separation is 1.5 $\leqslant G/D \leqslant$ 2. An enormous rise of harvested power was found by means of flat plate length *w*/*D* = 3 located *G*/*D* = 1.5 from the cylinder when the reduced velocity is ranging between 16 $\leqslant U_{R} \leqslant$ 19. The highest harvested power in this particular case was $P_{harv}$ = 9.627 $\times 10^{-2}$ W. However, the high harvested power was decreasing to very small magnitude power as the gap separation extended towards *G*/*D* = 3 in the third regime. This trend is similar to the case of *G*/*D* = 3 for plate length *w*/*D* = 0.5. The character of modified galloping was presumed in these cases based on the dynamic response analysis. As depicted by Fig. 29a, the magnitude of harvested power during the lower branch of velocity remained relatively low for all gap separations, which tend to be disadvantages for energy harvesting purpose.

The drawbacks of plate length *w*/*D* = 3 to harvest adequate power is explained by the plot of efficiency as shown in Fig. 29b. Although the power plots prevailed previously suggested that plate length *w*/*D* = 3 has an ample potential to generate high harvested power, due to high velocity requirement, the degree of effectiveness was diminished. For example, when two peaks of VIV lock-in for case of *G*/*D* = 2 were compared, the efficiency of power conversion was evidently decreased from $\eta _{\text {c-p}_{3}}$ = 5.9$\%$ to $\eta _{\text {c-p}_{3}}$ = 3.51$\%$ with the increased of reduced velocity. The shortfall of power conversion efficiency revealed in this case conveys the impracticality of long plate as the vibration enhancer for energy harvesting application. For reference, the maximum harvested power and its efficiency for different gap separation are recorded in Table 9.

**Table 9:** Summary of maximum harvested power and its efficiency for plate length *w*/*D* = 3 with different gap separation.

Gap separation	Reduced velocity	Wind velocity	Harvested power	Fluid power	Efficiency
*G*/*D*	$U_{R_{max}}$	*U*	$P_{harv_{max}}$	$P_{fluid}$	$\eta _{\text {c-p}_{3}}\%$
0.5	20	7.4	0.02291	1.99	1.15
1	18	6.7	0.00347	1.48	0.23
1.5	18	6.7	0.09627	1.48	6.50
2	17	6.3	0.04312	1.23	3.51
2.5	9	3.3	0.00457	0.18	2.54
3	9	3.3	0.00173	0.18	0.96

**Figure 30:**
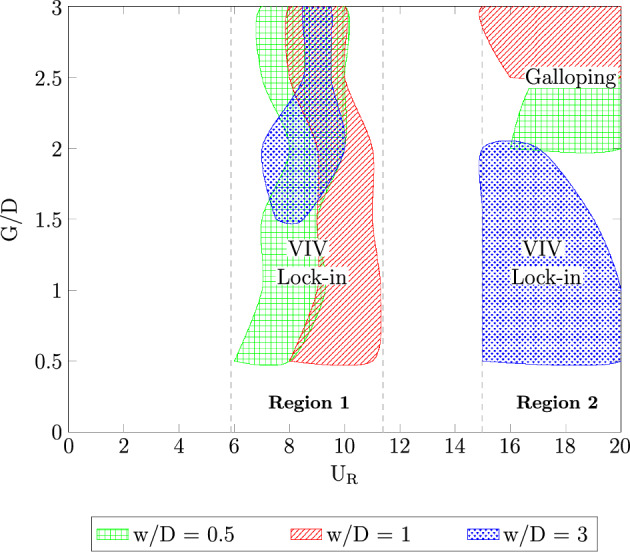
Map of energy harvesting for various configuration of cylinder-plate.

In this study, the harvested power magnitude was never intended for perpetual application. The harvested power is expected to be relatively lower than to the conventional and more established harvester due to its small inertial mass. Williams et al.^78^ suggested that the power output of below than 1$\mu$W is acceptable for low power application. In accordance to the results presented thus far, the minimum harvested power opted for benchmarking and generalization of this study was $P_{harv}$ = 0.3mW. Figure 30 illustrates the potential regions to harvest the energy by different configurations of cylinder-plate. According to the map, there are two main regions of which the estimated power is sufficient for energy harvesting purpose.

The power estimation is further explored by measuring the energy conserved relatively from the initial power of fluid and described as the efficiency of power conversion. The trend of efficiency for each plate lengths follows closely to the trend of harvested power estimation. For plate length *w*/*D* = 0.5, the efficiency of power conversion is consistently low in reference to the estimation of harvested power. Previously, the power curve plots have shown that plate length of *w*/*D* = 3 has successfully enhanced a remarkable magnitude of harvested power of a wider range of reduced velocity. Nevertheless, through the efficiency plots, flat plate length *w*/*D* = 1 overtakes the superiority of plate length *w*/*D* = 3. This was due to the high reduced velocity requirement to harvest substantially high power magnitude.

Based on the evaluation of the estimation of harvested power and its efficiency, flat plate of length *w*/*D* = 1 appeared to have a better reputation for harvesting airflow energy within low velocity range. Although the maximum power magnitude for the plate length *w*/*D* = 3 at gap separation *G*/*D* = 1.5 was higher than the maximum power for cases of plate length *w*/*D* =1, it was revealed that higher wind velocity is required to produce the substantial vibration and power. Taking the low wind speed condition of Malaysia into consideration, the first region (6 $\leqslant U_{R} \leqslant$ 12) was more suitable and relevant than the second velocity region. Besides, the degree of robustness for a harvester is also a major concern. In order to complement the design of a harvester for remote application, it is crucial to find an ideal flat plate length to reduce the size of the harvester while maintaining the sustainability of high amplitude throughout the lock-in synchronization regime. In accordance to the purpose of this study and the criteria of a robust harvester for low wind velocity application, the case of cylinder-plate of length *w*/*D* = 1 was opted for detailed investigation. Further investigation reckons the best location (gap separation) of flat plate from the square cylinder to harvest usable energy.

## Conclusion

Previous reports have revealed that the presence of plate downstream to a cylinder could alter the wake vortex dynamic and thus, change the character of flow-induced vibration experienced by the cylinder. Moreover, the character of FIV, which is determined by the dynamic response of cylinder is highly influenced by the length of a plate. In this study, three different flat plate lengths of *w*/*D* = 0.5, 1 and 3 were investigated. The prospect of the cylinder vibration amplitude enhancement by using these three flat plates was also assessed to determine the most efficient flat plate for energy harvesting purpose. Based on the numerical simulation results, the wake structure feature and dynamic character of vibration were affected not only by the variation of flat plate lengths *w*/*D* but also due to the gap separation between the square cylinder and the flat plate. Henceforth, the significant findings of each plate length cases are summarized.

For a cylinder-plate with length *w*/*D* = 0.5, two dynamic regimes were identified. The first regime is associated with relatively low amplitude vibration over reduced velocities trend for gap separation ranging between 0.5 $\leqslant G/D \leqslant$ 1.5. The small magnitude of vibration in this regime suggested the elimination of galloping towards the high branch of reduced velocity, which was found for the isolated cylinder case. The placement of flat plate in the wake of square cylinder at small gap separation has delayed the formation of fully developed vortex pair and consequently, reduced the vortex strength enforced unto the cylinder and resulted in the suppression of vibration amplitude. The second dynamic regime of cylinder-plate *w*/*D* = 0.5 is characterized by the coexistence of VIV and galloping during low and high branch of reduced velocity, respectively. The galloping character was confirmed through the frequency response and flow visualization of vorticity contours in the wake of cylinder. When the flat plate is located between 2 $\leqslant G/D \leqslant$ 3, the formation of a fully developed vortex pair can be found inside the gap separation between the cylinder and the flat plate.

Following the characterization of regimes determined for flat plate length *w*/*D* = 0.5, the first regime encapsulated cases of gap separation ranging between 0.5 $\leqslant G/D \leqslant$ 2 when the length of flat plate is *w*/*D* = 1. A remarkable high amplitude of VIV was established in the first regime when the gap separation ranging between 0.5 $\leqslant G/D \leqslant$ 1. Following that, in the same regime, the suppression of VIV amplitude during the lock-in synchronization was demonstrated. Moreover, a full mitigation of the galloping character in the higher branch of velocity was observed for the first regime. Besides that, through the flow visualization of vorticity contours, cases of different gap separation in the first regime also depicted the elongated vortex formation behind the flat plate. The consequent character of VIV and galloping in the amplitude curve over the considered range of reduced velocity occurred in the second regime, indicating the recovery trend of vibration amplitude similar to the case of an isolated square cylinder when the flat plate is located at gap separation between 2.5 $\leqslant G/D \leqslant$ 3. The significance of flat plate in controlling the wake flow is diminished in the second regime.

Unlike the two previous cases of different flat plate lengths, the findings for the case of plate length w/D = 3 was peculiar. Based on the evaluation of dynamic response, three dynamic regimes were obtained for case *w*/*D* = 3. The tendency of producing high vibration amplitude, which simultaneously increased the generated power reclined towards the higher branch of velocity in the first regime. This character was evident for cases 0.5 $\leqslant G/D \leqslant$ 1. The second regime revealed dual peaks of VIV lock-in synchronization when the separation distance of cylinder and plate was 1.5 $\leqslant G/D \leqslant$ 2. However, with further extension of gap separation to *G*/*D* = 2.5, the magnitude of vibration amplitude was dramatically collapsed. Finally, when the plate is placed at *G*/*D* = 3 downstream to the cylinder, the second peak of VIV was fully mitigated and only the first peak remained. The suppression of vibration amplitude in the third regime particularly towards the higher branch of reduced velocity is due to growth of vortex formation length caused by the decreased of base suction and Reynolds stress.

By utilizing the vibration amplitude results, the harvested power estimation of different flat plate length was calculated. The prospect of energy harvesting is illustrated in Fig. 30 with the minimum harvested power of $P_{harv}$ = 0.3mW. In general, for all cases, the lock-in synchronization of VIV was enclosed in the range 6 $\leqslant U_{R} \leqslant$ 12. For flat plate of *w*/*D* = 0.5 and 1, the galloping phenomenon occurred during the high velocity starting from $U_{R} \geqslant$ 15. The galloping occurence in this particular case contributed to the remarkable high amplitude and estimated power. Contrarily, for case of *w*/*D* = 3, the second region of VIV synchronization was observed during the higher velocity branch. The second region of VIV was revealed when the gap separation between cylinder and plate varied between 0.5 $\leqslant G/D \leqslant$ 2. The vibration amplitude and estimated power increased throughout this region particularly when the gap separation between cylinder and flat plate was *G*/*D* = 1.5.

According to the evaluation of harvested power and its efficiency of each plate length case, the prospect of harvesting wind energy by utilizing the cylinder-plate of length *w*/*D* = 1 is impressive. Taking the low wind speed limitation and the concern of harvester’s robustness, plate of such length when placed downstream of elastic square cylinder at gap distance *G*/*D* = 1 is able to produce maximum harvested power $P_{harv}$ = 0.03827 W for reduced velocity $U_R$ = 11 (*U* = 4.5 ms$^{-1}$). The vibration of amplitude for single square cylinder has successfully increased from $y_{rms}$ = 0.005 to $y_{rms}$ = 0.14 by wake modification from the flat plate. Since the vibration amplitude is significantly influenced the amount of harvested power, it is justified that production of harvested power by cylinder-plate configuration is also increased.

## Data Availability

The datasets used and/or analysed during the current study available from the corresponding author on reasonable request.
